# Isoproterenol mechanisms in inducing myocardial fibrosis and its application as an experimental model for the evaluation of therapeutic potential of phytochemicals and pharmaceuticals

**DOI:** 10.1002/ame2.12496

**Published:** 2024-12-17

**Authors:** Lujain Bader Eddin, Mohamed Fizur Nagoor Meeran, Niraj Kumar Jha, Samer N. Goyal, Shreesh Ojha

**Affiliations:** ^1^ Department of Pharmacology and Therapeutics, College of Medicine and Health Sciences UAE University Al Ain United Arab Emirates; ^2^ School of Bioengineering & Biosciences Lovely Professional University Phagwara India; ^3^ Centre for Global Health Research, Saveetha Medical College, Saveetha Institute of Medical and Technical Sciences, Saveetha University Chennai India; ^4^ Shri Vile Parle Kelvani Mandal's Institute of Pharmacy Dhule Maharashtra India; ^5^ Zayed Bin Sultan Center for Health Sciences United Arab Emirates University Al Ain United Arab Emirates

**Keywords:** cardiac fibrosis, catecholamines, experimental models, isoproterenol, myocardial fibrosis, phytochemicals, β‐adrenergic receptors

## Abstract

Cardiac injury initiates repair mechanisms and results in cardiac remodeling and fibrosis, which appears to be a leading cause of cardiovascular diseases. Cardiac fibrosis is characterized by the accumulation of extracellular matrix proteins, mainly collagen in the cardiac interstitium. Many experimental studies have demonstrated that fibrotic injury in the heart is reversible; therefore, it is vital to understand different molecular mechanisms that are involved in the initiation, progression, and resolution of cardiac fibrosis to enable the development of antifibrotic agents. Of the many experimental models, one of the recent models that has gained renewed interest is isoproterenol (ISP)–induced cardiac fibrosis. ISP is a synthetic catecholamine, sympathomimetic, and nonselective β‐adrenergic receptor agonist. The overstimulated and sustained activation of β‐adrenergic receptors has been reported to induce biochemical and physiological alterations and ultimately result in cardiac remodeling. ISP has been used for decades to induce acute myocardial infarction. However, the use of low doses and chronic administration of ISP have been shown to induce cardiac fibrosis; this practice has increased in recent years. Intraperitoneal or subcutaneous ISP has been widely used in preclinical studies to induce cardiac remodeling manifested by fibrosis and hypertrophy. The induced oxidative stress with subsequent perturbations in cellular signaling cascades through triggering the release of free radicals is considered the initiating mechanism of myocardial fibrosis. ISP is consistently used to induce fibrosis in laboratory animals and in cardiomyocytes isolated from animals. In recent years, numerous phytochemicals and synthetic molecules have been evaluated in ISP‐induced cardiac fibrosis. The present review exclusively provides a comprehensive summary of the pathological biochemical, histological, and molecular mechanisms of ISP in inducing cardiac fibrosis and hypertrophy. It also summarizes the application of this experimental model in the therapeutic evaluation of natural as well as synthetic compounds to demonstrate their potential in mitigating myocardial fibrosis and hypertrophy.

## Graphical abstract

A summary of ISP mechanisms in inducing cardiac fibrosis.

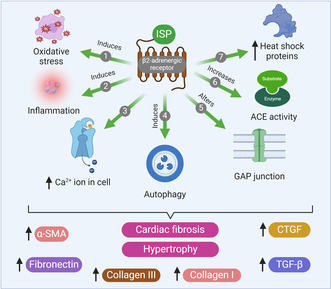

## 1 INTRODUCTION

Myocardial fibrosis is a scarring process that occurs in cardiomyocytes, where accumulated extracellular matrix (ECM) proteins impact the quality and impair the function of myocytes. Cardiac fibrosis is characterized by the excessive deposition of collagen type I released by the activated cardiac fibroblasts: the myofibroblast. Myofibroblasts also release growth factors, inflammatory cytokines, and collagen‐synthesizing enzymes.^1^ Hypertrophy is a subsequent consequence of cardiac fibrosis that is represented by an enlarged and thickened heart tissue. Cardiac fibrosis occurs in response to exposure to ischemic injury, chronic hypertension, and aging. Despite the self‐healing ability of human body cells including cardiomyocytes, not all injured tissues can be properly regenerated. Myocytes are prone to damaging defects, preventing the ideal remodeling process that can end up in the buildup of collagen compensating for dead cells.^2^ The compensating collagen type I has been found to form four types of fibrotic structures: interstitial, compact, diffuse, and patchy. Interstitial fibrosis is characterized by the accumulation of collagen between cell groups, compact fibrosis by the formation of large and dense fibrous structures, diffuse fibrosis by short stretches of collagenous structures, and patchy fibrosis by long strands of collagen.^3^ Reasonably produced collagen is beneficial for preserving myocardial integrity. However, overproduction of collagen causes cardiac dysfunction by limiting cardiac contractility and relaxation and impairing electrical coupling where the cross‐linked fibers act as electric insulators.^4^

Cardiac fibrosis can be experimentally produced using surgical models and genetic models or can be chemically induced. Many experimental studies have demonstrated that fibrotic injury in the heart is reversible; therefore, it is vital to understand the different molecular mechanisms that are involved in the initiation, progression, and resolution of cardiac fibrosis to enable the development of antifibrotic agents. Of the many experimental models, one of the recent models that has gained renewed interest is the induction of myocardial fibrosis by activating β‐adrenergic receptors through isoproterenol (ISP) administration. The sustained and overstimulated β‐adrenergic receptors have been reported to cause cardiac remodeling. ISP has been used for decades to induce acute myocardial infarction. However, the use of low doses and chronic administration of ISP have been shown to induce cardiac fibrosis; this practice has been increasing in recent years. ISP has been widely used to induce cardiac fibrosis pathogenesis both acutely and chronically.

ISP is a synthetic, sympathomimetic, nonselective β‐adrenergic agonist, chemically known as 4‐[1‐hydroxy‐2‐[(1‐methyl ethyl)amino]ethyl]‐1,2‐benzenediol, monohydrochloride or isoprenaline, used in the treatment of brady‐dysrhythmias.^5^ It has been known to induce myocardial infarction–like lesions in laboratory rats and is widely used for experimentally evaluating compounds for their cardioprotective properties. It has been reported that ISP produces free radicals that induce oxidative stress and alter the biochemical balance of the heart. This alongside other cellular mechanisms impairs cellular integrity, distorts cardiomyocyte histology, and triggers molecular mechanisms that cause perturbations at the transcriptional level altering cellular homeostasis and predisposing cellular demise. Therefore, ISP‐induced myocardial injury has been utilized in research as a screening model that resembles the pathological features of cardiac fibrosis. ISP has been used as an inducing agent for both acute and chronic cardiac damage; it can cause progressive damage when administered consecutively in fractionated doses. Therefore, the ISP‐induced myocardial injury model enables researchers to evaluate the potential cardioprotective compounds and the possible underlying mechanisms.^6^ The present review provides an insight into the pathological, biochemical, molecular, and cellular mechanisms of ISP‐induced cardiac fibrosis and discusses the usage of ISP‐induced myocardial fibrosis as an important experimental model to evaluate the potential therapeutic agents. The dosage regimen of ISP used in different experimental models and the observed mechanisms of ISP to induce cardiac remodeling are summarized in Table 1.

**TABLE 1:** Dosage regimen of ISP used in different experimental animals and observed mechanism of myocardial fibrosis. Abbreviations: ACE, angiotensin‐converting enzyme; ANP, atrial natriuretic peptide; AKT: serine/threonine kinase known as protein kinase B; BNP, brain natriuretic peptide; Cx43, connexins 43; Gal‐3, galectin‐3; HR: Heart rate; HSP, heat shock protein; IL, *interleukin*; i.p., intraperitoneal; ISP, isoproterenol; LKB1IP, liver kinase B1 interacting protein 1; LVEDV: Left Ventricle Enddiastolic Volume; LVFS: Left Ventricle Fractional shortening; LV: Left Ventricle; LVW/TL: Left ventricle weight//tibial length; Ly6c: Lymphocyte antigen 6 complex; mPGES‐1, microsomal prostaglandin E synthase‐1; MCP‐1: Monocyte chemoattractant protein‐1; mRNA, messenger RNA; NADPH, nicotinamide adenine dinucleotide phosphate; PARP‐1, poly(ADP‐ribose)polymerase 1; P.o: Orally; RV: Right ventricle; s.c., subcutaneous; SD, Sprague–Dawley; SIRT1, sirtuin 1; SMA, smooth muscle actin; STAT6, signal transducer and activator of transcription 6; TFEB, transcription factor EB; TXNDC5, thioredoxin domain containing 5; β‐MHC, β‐myosin heavy chain.

ISP dosing regimen and species	Mechanisms/effects	Reference
300 mg/kg, s.c. twice for two consecutive days to transgenic mice	↓LVFS, ↑LV diameter, ↑MCP‐1, ↑LVW/TL, ↑Gal‐3, CD68	[7]
100 mg/kg/day, s.c. for 5 days to male Swiss Webster mice	↑Collagen I and III, ↓viable cells, ↓LV compliance, ↓LV and RV weight	[8]
60 mg/kg/day for 14 days by osmotic minipump and miR‐133a transgenic mice	↑microRNA‐133a	[9]
5 mg/kg/day, s.c. for 10 days to male SD rats	↑Collagen, ↑ACE activity ↑ACE mRNA	[10]
40 mg/kg on day 1, 20 mg/kg on day 2, or 10 mg/kg on day 3, and 5 mg/kg s.c. on day 4 and continued for 10 days to HSF‐1 knockout mice	↑Collagen, ↑HSF‐1, ↑HSP47	[11]
10 mg/kg/day, s.c. for 14 days to LKB1IP knockout and C57BL/6J mice	↑ANP, BNP, and β‐MHC ↑mLKB1IP, ↑AKT	[12]
15 mg/kg/day for 11 days by osmotic minipump to male C57BL/6 mice	↑HR, ↑heart mass, ↑collagen ↑superoxide, NADPH	[13]
30 mg/kg/day, s.c. for 10 days to C57BL/6J male mice	↑TXNDC5, ↑TGF‐β1	[14]
5 mg/kg/day, s.c. for 7 days to male Wistar rats	↑Urotensin II, ↑urotensin II receptor ↑angiotensin II ↑collagen	[15]
5 mg/kg/day for seven consecutive days to C57BL/6, mPGES‐1 knockout mice	↑mPGES‐1, ↑collagen I, collagen III, fibronectin	[16]
85, 100, and 120 mg/kg/day once, s.c. for 2 days to male SD rats	↑Fibrotic region, ↑α‐SMA ↑collagen I and III	[17]
5 mg/kg/day, s.c. for 10 days to SD rats	↑TRPM7, ↑collagen I, ↑α‐SMA, ↓miR‐135a	[18]
60 mg/kg/day, for 14 days by osmotic minipump to MycCre+, WP2^Fl/Fl^ and MycCre−, and WWP2^Fl/Fl^ mice	↑PARP‐1, PARylation	[19]
5 mg/kg/day, s.c. for 7 days to C57BL/6 mice	↑IL‐18, ↑macrophage	[20]
10 mg/kg body, s.c., single injection to SD rats	↑fM1/M2 polarization of macrophage	[21]
6 mg/kg/day, s.c. for seven consecutive days to C57BL/6 mice	↑LC3‐II and P62, ↑autophagosome ↓SIRT1/TFEB, ↓SIRT1/P53	[22]
5 mg/kg, i.p. for 4 weeks to BALB/C mice	↓STAT6, ↑CD11b^+^ myeloid cells ↑CD11b^+^Ly6C^+/low^ macrophage ↑IL‐1α, IL‐18, and TGF‐β1	[23]
5 mg/kg, s.c. for 7 days to SD rats	↓Cx43	[24]

ISP formulations are used to induce myocardial fibrosis and hypertrophy. Given the need to mimic the advanced stages of heart fibrosis and failure, ISP has been formulated as an implanted mini‐osmotic pump. ISP leads to a gradual and progressive cardiac damage if administered in fractionated doses. Thus, ISP constantly stimulates the adrenergic receptors, which are relatively simple and reproducible to recapitulate the late stages of heart fibrosis. The continuous administration of ISP using a minipump over 4 weeks was reported to efficiently induce cardiac remodeling^25^; 30 mg/kg/day of ISP infused intraperitoneally using a mini‐pump for 21 days led to a striking shift in heart fibrotic parameters from normal levels to a significant change in their expression when compared with the control group.^26^ Many other studies reported the subcutaneous administration of 5–10 mg/kg of ISP for 7–14 days to induce cardiac fibrosis. Intraperitoneal administration of ISP to induce cardiac fibrosis has also been reported. The doses used are relatively similar to those administered subcutaneously with the same duration.

The different dosing regimens used in different animal species and cell lines are presented in Table 1, summarizing the doses and duration used for ISP for inducing cardiac fibrosis in experimental studies. It has been documented that male rodents are preferred to females for the induction of fibrosis and hypertrophy as they are more susceptible to fibrosis induction due to the possible increased activation of fibroblasts in male heart compared with the female heart^27^ with increased gene expression of adrenergic cascade mediators^28^ as well as increased expression of both β1‐ and β2‐adrenergic receptors.^29^ However, an equal propensity to developing cardiac fibrosis is reported in both males and females with similar induction of fibrotic genes. The study emphasized the lack of sexual dimorphism indicated by the absence of differences in the degree of fibrosis induction after gonadectomy in both sexes.^30^ The pharmacologically induced cardiac remodeling using ISP is considered a simple model as it does not involve surgical procedures or the expenses of genetic‐based models.

Obviously, it has been extensively used in studies screening for potential therapeutic candidates for cardiac remodeling, as summarized in Tables 2, 3, 4, 5, which present all phytochemicals as well as synthetic compounds that have been evaluated for their protective effect against cardiac remodeling. Many studies demonstrate that mouse‐based models are preferred and suitable for studying the pathogenesis of cardiac fibrosis and hypertrophy and evaluation of therapeutic or preventive agents, as presented in Table 2. However, rats are preferred for ISP‐induced myocardial necrosis resembling MI.

**TABLE 2:** Phytochemicals exhibited therapeutic potential in ISP‐induced myocardial remodeling and fibrosis in murine models.

Phytochemical (plant)	Compound dosing	ISP dosing regimen and species	Effects and mechanisms	Reference
Plantamajoside (*Herba Plantaginis*)	20, 40, and 80 μmol/L for 24 h 10 and 40 mg/kg/day, i.v. 3 days before ISP continuing for 9 days	10 μmol/L to H9c2 cells, 5 mg/kg/day s.c. for 9 days to BALB/c mice, 3 days after Plantamajoside treatment	↓Cell surface area, embryonic proteins, and genes ↓LVPW LVAW, and LVID ↑EF, FS ↓HW/BW, LVW/BW, HW/tibia length ↓Irregular morphology, disordered arrangement and fibroblast proliferation, and collagen fibers, ↓ANP, BNP, Myh7, COL1 and COL3, p‐HDAC2, p‐AKT, and p‐GSK3β	[31]
Xanthohumol (*Humulus lupulus L.)*	1 mg/kg/day for 14 days p.o.	5 mg/kg, twice a day for 14 days, s.c. to old male C57BL/6 mice (6–8 weeks)	↑EF%, FS% ↑PTEN ↓CK and cTnT, ANP, BNP, collagen, α‐SMA, p‐AKT, p‐mTOR	[32]
Resveratrol (grapes, peanuts)	20 mg/kg/day for 14 days, i.p. injection	10 mg/kg/day for 3 or 4 days and then 5 mg/kg/day for 11 days, s.c. injection to male C57BL/6 mice	↓Left ventricular mass ↑LVIDd, LVIDs, LVEDV ↓α‐SMA, LVESV, myofibril disarray, collagen deposition ↓Collagen I and collagen III, TGF‐βR1, P‐Smad‐2/3 ↑SIRT1	[33]
Piperine (*Piper longum*)	50 mg/kg/day, oral gavage for 3 weeks	50 mg/kg for 14 days, s.c. to male C57/B6 mice (8–10 weeks)	↓Collagen, ↓α‐SMA ↑PPAR‐γ	[34]
Amlexanox (ALX) and Forskolin (FSK) (*Coleus forskohlii*)	ALX: 2.5 mg/100 g, i.p., FSK 0.5 mg/100 g, i.p.	0.5 mg/100 g/day, s.c. to FVB mice for 40 days	↑βARs, AC5, and AC7 ↓AC6, β‐ARR‐1, β‐ARR‐2 GRK2, and GRK5 ↓GATA4, NFAT ↓cAMP, MEF2, and NF‐κB ↓ANP, BNP ↓IL‐1β, IL‐6, TNF‐α	[35]
ALX and FSK (*Coleus forskohlii*)	2.5 mg/100 g/day, i.p. and 0.5 mg/100 g/day, i.p.	0.5 mg/100 g/day of ISP, s.c. to FVB male mice for 21 days	↓CD86+ infiltration ↑EF, FS, ↓LVSD, ↓ANP, BNP, ↓apoptosis, ↓collagen I and III ↑CD206+ ↓iNOS, IL‐1β, IL‐6, TNF‐α ↓TGF‐β1, IL‐10, GPK5 ↑cAMP	[36]
inomenine *(Sinomenium acutum)*	120 mg/kg p.o. for 28 days	40 mg/kg on day 1, 20 mg/kg on day 2, 1 mg/kg for 26 days s.c. to C57BL/6J male mice	↓NF‐κB, LDH, MDA, TNF‐α, and IL‐1β, ↑SOD	[37]
Triptolide (*Tripterygium wilfordii* Hook F)	10, 30, and 90 μg/kg i.p. for 14 days	5 mg/kg/day s.c. for 14 days to mice	↓HW/BW, LVW/BW, W/TL, and LVW/TL, ↓β‐MHC, ANP, cTnI, ↓fibrosis, necrotic tissue, ↑Foxp3	[38]
Gallic acid (berries, grapes, tea, etc.)	100 mg/kg, i.p. 1 week before ISP and an additional 2 weeks	25 mg/kg/day, osmotic minipump for 2 weeks to male CD‐1 (ICR) mice	↓Smad‐3, ANP, BNP, MHC ↓Collagen I and III, ↓p‐c‐JNK, ↓ERK, ↓GATA4	[39]
Periplocymarin (*Periplocae cortex*)	5 mg/kg, s.c. for 7 days	5 mg/kg, s.c. for 7 days to C57BL/6 mice	↓Col1a1, Col3a1, Acta2, Tgfb1 ↓Collagen I, collagen III, α‐SMA and TGF‐β1, COX‐2	[40]
Stevioside (*Stevia rebaudiana*)	75, 150, and 300 mg/kg, p.o. once daily for 40 days	5 mg/kg, s.c., reduced to 2.5 mg/kg and for 30 days to mice	↓Collagen I, collagen III, α‐SMA, hydroxyproline ↑SOD and GSH‐PX, PPAR‐γ ↓NF‐κB p65 and TGF‐β1, Smad‐2/3 and P‐Smad‐2/3	[41]
Isoglycyrrhizinate (*Glycyrrhiza glabra*)	25 mg/kg and 50 mg/kg, i.p. for 2 weeks	5 mg/kg, s.c., twice a day for 14 days to mice	↓ANP, BNP, c‐fos, c‐jun, and α‐MHC mRNA, ↓Bax ↓NF‐κB (p65), CK and LDH	[42]
Cryptotanshione (*Salvia miltiorrhiza*)	20 mg/kg, p.o. for 2 weeks	3 mg/kg, s.c. for 2 weeks to C57BL/6 mice	↓MMP‐2	[43]
Baicalein (*Scutellaria baicalensis* Georgi)	25 mg/kg, i.v. on days 3, 6, 9, 12, and 15	30 mg/kg, i.p. for 15 days to C57BL/6, mice	↑Catalase, ↑LC3‐II/I, p62 ↑Autophagosomes, ↑FUNDC1 ↑FOXO3a	[44]
Shikonin	2 or 4 mg/kg, p.o. for 21 days	5 mg/kg, s.c., for 7 days to C57BL6 mice	↓TGF‐β1, MMP‐9, TLR‐9, CD14, IL‐1b, TNF‐α, IL‐6, IL‐12, TLR4, MyD88, NF‐κB ↑Bcl‐2, Mcl‐1 ↓GRP78, p‐PERK, p‐eIf2a, IRE1, ATF6	[45]
Notoginsenoside R1	1–50 mg/kg, i.p. for 7 days	25 mg/kg, osmotic minipumps for 14 days to ApoE−/− C57BL/6J mice	↓α‐SKA, MMP‐9, Ly6C^high^ monocytes ↓TNF‐α, MCP‐1, IL‐1β, IL‐6, CCR2	[46]
Crocin	200 mg/kg, i.p. for 14 days	5 mg/kg, s.c. twice a day for 14 days to Kunming mice	↓LDH and CK ↑SOD, CAT, GSH ↓IL‐6, TNF‐α, NF‐κB (p65), TLR4, Bcl‐2‐associated X protein, caspase‐3 ↑Bcl‐2 ↓TGF‐β1, CTGF	[47]
Luteolin‐7‐diglucuronide	5–40 mg/kg, i.p. for 5 days	5 mg/kg, i.p. for 5 days to C57BL/6J mice	↓Cyba, Cybb, Ncf1, Ncf4, and Rac2	[48]
Withaferin A	4 mg/kg, i.p. for 14 days	50 mg/kg, s.c. for 2 days to mice	↓α‐SMA, collagen I, III	[49]
Genistein	100 mg/kg in diet, pretreatment for 7 days and continued for 14 days	30 mg/kg for 14 days to mice	↓miR‐199, miR‐499 ↑miR‐133, miR‐451 ↓TIMP‐2	[50]
Apocynin	12.5, 25, 50, 100 mg/kg, i.p.	5 mg/kg, i.p. for 5 days to 57BL/6 J mice	↓ROS, NADPH oxidase ↓p‐Smad‐2, TIMP‐1, and TIMP‐2	[51]
Evodiamine	50 and 100 mg/kg, p.o. for 14 days	10 mg/kg s.c. for 3 days and 5 mg/kg for 11 days to C57BL/6 mice	↓Collagen I, III, CTGF, TGF‐β1, fibronectin, ↓EndMT ↓CD34 and CD31	[52]
Cycloastragenol	100 or 200 mg/kg/day, p.o. for 7 days	5 mg/kg, s.c. for 7 days to BALB/c mice	↓NLRP3, caspase‐1, IL‐18, and IL‐6	[53]
Qiligiangxin	0.5 g/kg, p.o. for 14 days	60 mg/kg, i.p. for 14 days to C57/BL6 mice	↓Bax, ↑Bcl‐2, ↓TGF‐β, MMP‐9 and MMP‐2, ↑PPAR‐γ and PGC‐1α	[54]
Engeletin	10, 25, and 40 mg/kg, i.p. for 14 days	10 mg/kg for 14 days to C57BL/6 mice	↑Cx43, Nrf2/HO‐1 ↓CK‐MB and LDH	[55]
Hydroxysafflor yellow A	100 mg/kg, p.o. for 2 weeks	5 mg/kg, s.c. for 2 weeks to C57BL/6 mice	↓L‐1β and IL‐18 ↓ASC ↓NLRP3 inflammasome	[56]
Mogroside IIIE	1 and 10 mg/kg, p.o. for 2 weeks	40 mg/kg, s.c. on day 1, 20 mg/kg on day 2, 10 mg/kg on day 3, and 5 mg/kg on day 4; 5 mg/kg used for another 10 days to C57BL/6 mice	↓TLR4, MyD88, and p‐NF‐κB‐p65	[57]
Gypensapogenin I	10, 20, and 40 mg/kg, p.o. for 21 days and 5, 7.5, and 10 μM to H9c2 cells	10 mg/kg, intrahepatic injection to C57BL6 mice and 50–300 μM for 24 and 48 h to H9c2 cardiomyocytes	↑Bcl‐2/Bax, ↓caspase‐3 and PARP‐1, ↓TLR4/NF‐κB/NLRP3, MyD88, and NF‐κB SOD, CAT, GSH‐PX	[58]
Syringic acid	100 mg/kg 7 days before ISP infusion and cotreated for 5 days with ISP and 10 μM acid for 24 h to H9c2 cells	25 mg/kg by osmotic minipump for 5 days to CD‐1 mice and 10 μM acid for 24 h to H9c2 cells	↓Nppa, Nppb, Col1a1, and Fn1 ↓Ereg, Ngfr, and Myc	[59]
8‐Gingerol	10 and 20 mg/kg, i.p. for 14 days	10 mg/kg, s.c. for 14 days to Kunming mice	↓ROS, ↓autophagosome ↑PI3K/Akt/mTOR	[60]
Liquiritigenin	10, 20, and 30 mg/kg for 2 weeks	5 mg/kg, s.c. on day 1, followed by 2.5 mg/kg for 2 weeks to C57BL/6 mice	↓TGF‐β1/Smad‐2 ↓AKT/ERK	[61]
Forsythiaside B	5 and 10 mg/kg, i.p. for 21 days	5 mg/kg, s.c. on day 1, and 2.5 mg/kg from days 2 to 21 to BLAB/c mice	↓TGF‐β	[62]

Abbreviations: AC5: Adenylyl Cyclase isoform 5; AC6: Adenylyl Cyclase isoform 6; AC7: Adenylyl Cyclase isoform 7; ANP, atrial natriuretic peptide; βARs: Beta asrenergic receptors; βARs: Beta asrenergic receptors; β‐ARR‐1: #x003B2;‐arrestin‐1; β‐ARR‐2: β‐arrestin‐2; BNP, brain natriuretic peptide; BW: body weight; CK, creatine kinase; CK‐MB, creatine kinase isoenzyme; cTnI: Cardiac Troponin I; Cx43, connexins 43; EF, ejection fraction; EndMT, endothelial‐to‐mesenchymal transition; FS, fractional shortening; FUNDC1: FUN14 Domain Containing 1; FVB: Ventricular Fibrillation Mice; GATA4: GATA‐binding protein 4; GRK2: G‐protein‐coupled receptor kinase 2; GRK5: G‐protein‐coupled receptor kinase 5; GPK5, G‐protein‐coupled receptor kinase 5; GSH, glutathione; GSK3β, glycogen synthase kinase‐3β; GSH‐PX: glutathione peroxidase; HW: heart weight; IL, *interleukin*; i.p., intraperitoneal; ISP, isoproterenol; i.v., intravenous; LDH, lactate dehydrogenase; LVEDV: Left Ventricle End‐diastolic Volume; LVESV: Left Ventricle End‐systolic Volume; LVPW: left ventricular posterior wall; LVAW: left ventricular anterior wall; LVID: left ventricle internal diameter; LVSD: Left ventricular systolic dysfunction; MDA, malondialdehyde; MEF2, myocyte enhancer factor‐2; MMP‐9, matrix metalloproteinase‐9; mRNA, messenger RNA; mTOR, mammalian target of rapamycin; MYD88: Myeloid differentiation primary response 88; NADPH, nicotinamide adenine dinucleotide phosphate; NF‐κB, nuclear factor kappa B; NFAT: Nuclear factor of activated T cells; PARP‐1, poly(ADP‐ribose)polymerase 1; PGC‐1α, proliferator‐activated receptor gamma coactivator 1‐alpha; p‐P.o: Orally; PPAR‐γ, peroxisome‐proliferator activated receptor‐γ; PTEN, phosphatase and tensin homolog; s.c., subcutaneous; SIRT1, sirtuin 1; SMA, smooth muscle actin; SOD, superoxide dismutase; TLR4, toll‐like receptor 4; TNF‐α, tumor necrosis factor‐α; α‐MHC, α‐myosin heavy chain.

Experimental models used for understanding pathogenesis or evaluating preventive and therapeutic agents often rely on rapid onset with a single stimulant or challenges and produce only some features of fibrosis. However, in humans the patterns of fibrosis, hypertrophy, scar, inflammation, edema, and apoptosis are multifactorial in nature. ISP‐induced myocardial fibrosis often results from sustained activation of adrenergic receptors due to the chemical nature of ISP itself. The subcutaneous, intraperitoneal injections and delivery by osmotic pumps consequently reproduce many pathogenic features of the cardiac fibrosis phenotype. One of the major issues in cardiovascular disease pathogenesis and diagnosis is the complex and multifactorial nature of the diseases.

Both rats and mice have many benefits such as easy in handling, housing, breeding due to a short duration, and maintenance. The dose of the test agent required (in mg/kg) is more for mice compared to rats, but due to the small size and weight of mice, adult mice corresponding to the same age of adult rats are 10 times less in weight, and therefore require lesser amount of the drug and are inexpensive. The surgical aspects such as intubation, ventilation, organ access, and relatively bigger organ size as well as the volume of body fluids in rats provide benefits over mice. The surgical techniques, whether catheterization, ligation, or occlusion, are relatively easier in rats than in mice. Rats are also considered closer to mammals and humans due to a relatively better symmetry of cardiovascular anatomy, physiology, and energetic pathways. Both rats and mice have a high heart rate, a shorter cardiac cycle, and slight differences in proteins such as α‐myosin in rats compared to β‐myosin heavy chains (β‐MHC) in humans. These differences in physiological and biochemical correlates, including cellular bioenergetics and mitochondrial function, play a role in translating the efficacy observed in animals into humans. Most of the available literature on animal models of ISP‐induced fibrosis suggests the mouse model to be a preferred choice in cardiac remodeling research. The reasons could be due to its small size, ease in handling, a short gestational period, and the ability to use a large number of animals without concerns raised by the animal protection and ethics committees as reported previously.^111^

## 2 ISP INDUCES CARDIAC FIBROSIS AND HYPERTROPHY

**FIGURE 1:**
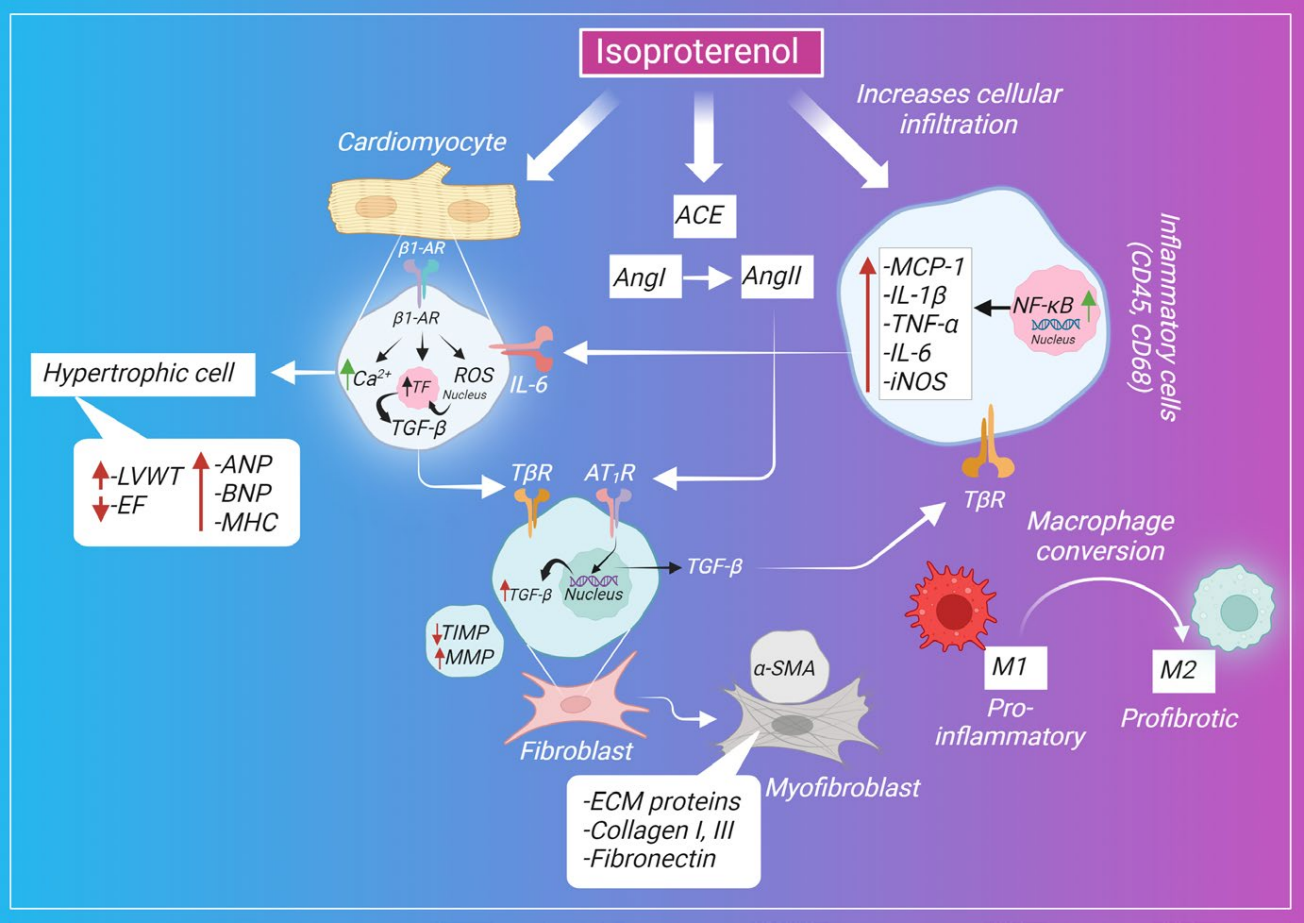
ISP (isoproterenol) effect on activating cardiomyocytes, fibroblasts, and inflammatory cells and inducing cardiac fibrosis.

Catecholamines or adrenergic agonists increase the contractility force and the heart rate, leading to an increase in cardiac output and oxygen consumption. In turn, the excess levels of catecholamines in blood circulation, whether endogenously released or exogenously administered, can eventually cause cardiomyocyte remodeling and myocardial damage.^112^ ISP is a synthetic nonselective β‐adrenoceptor agonist that has been used in research for developing models mimicking the pathological features of cardiac fibrosis and hypertrophy intended to reveal the underlying cellular and molecular mechanisms implicated in their pathogenesis and enabling researchers to investigate the cardioprotective role of various plant extracts, phytochemicals of natural origin, and pharmaceutical compounds.^63^ The proposed effect of ISP on various cells that mediate cardiac fibrosis is shown in Figure 1.

The sustained activation and overstimulation of β‐adrenergic receptors result in cardiac remodeling as a result of hemodynamic overload and increased cardiac workload. In 1973 the first report was published on ISP demonstrating its role in causing myocardial hypertrophy, increased ventricular weight, and cardiac fibrosis.^113^ The administration of ISP alters the dynamics of β‐receptors through the constant activation of β‐receptors controlling heart contractility, resulting in increased cardiac preload and afterload, cellular loss, cardiac remodeling as a compensatory mechanism for necrotic cells, and ultimately heart failure.^112^ Several mechanisms, including fibroblast activation, oxidative stress induction, impaired mitochondrial bioenergetics, sustained inflammatory response, calcium ion dysregulation, and alteration in the transcriptional level of the fibrotic gene, have been reported to be implicated in ISP‐induced cardiac fibrosis and hypertrophy.

### 2.1 Cellular mechanisms

#### 2.1.1 ISP induces expression of fibrotic proteins

Cardiac collagen accumulation along with extracellular matrix proteins in the cardiac interstitium is the major feature of cardiac fibrosis.^114^ The fibrotic‐induced effect of ISP was assessed by quantifying Masson's trichrome staining in most studies. It is reported that ISP caused an increase in the fibrotic region (blue‐stained areas) compared with the control group, with an increase in the deposition of collagen I and III in the left atrium compared with the antifibrotic pirfenidone drug. α‐Smooth muscle actin (α‐SMA) expression is the feature of mature fibroblasts. ISP induces the differentiation of fibroblasts into myofibroblasts evident by the stained α‐SMA that contributes to the fibrotic process.^17^

ISP‐induced cardiac fibrosis was further confirmed by the detection of the fibrosis‐related molecules. ISP induces a remarkable increase in the expression of collagen I and III, laminin, TGF‐β1, and α‐SMA. A similar study showed that ISP triggers the activation of nuclear factor kappa B (NF‐κB); mitogen‐activated protein kinases (MAPK); and its downstream kinases such as p38, ERK, and JNK that are known to be involved in the aggravation of fibrosis.^103^ This is consistent with the recent finding that demonstrated the effect of β‐adrenergic receptor activation on fibroblast proliferation through Gαs‐mediated activation of ERK1/2 signaling.^115^ The inhibition of p38MAPK and ERK1/2 was clearly correlated with fibroblast proliferation suppression.^116^ Another study showed that ISP was found to phosphorylate MAPK signaling mediators, including p38, ERK, and JNK, causing a significant induction in fibroblast activation and proliferation.^106^

Fibroblast cells produce a number of cytokines, peptides, and enzymes such as tissue inhibitor of metalloproteinase‐1 (TIMP‐1) and matrix metalloproteinase‐9 (MMP‐9), for which any imbalance in their activities affects the extracellular matrix turnover and homeostasis.^117^ ISP downregulates TIMP‐1 and upregulates MMP‐9, which is responsible for cleaving the extracellular matrix and fibrosis propagation.^103^ Similarly, the fibrotic‐linked gene expressions such as *MMP‐2, MMP‐9, TGF‐β1, fibronectin, α‐SMA, collagen I, collagen III, Smad‐2, Smad‐3, TIMP‐2, angiotensin II receptor, CTGF, Endothelin‐1(ET‐1), Activating Protein‐1(AP‐1),  Intercellular adhesion molecule (ICAM‐1), Vascular Cell Adhesion Molecule‐1 (VCAM‐I), E‐selectin, p38, JNK, ERK, ß‐catenin, peroxisome‐proliferator activated receptor‐γ (PPAR‐γ), and myocardin‐related transcription factor (MRTF)* were upregulated in the ISP‐induced heart, whereas *TIMP‐1, p‐AKT, p‐GSK‐3β,* and *PPAR‐γ* genes were downregulated.^68^

A significant increase in TGF‐β1, Smad‐2/3, and Smad‐4 was also reported previously in the model of ISP‐induced hypertrophy.^67^ Investigations also revealed that ISP induces the expression of fibrosis‐related genes *Col1a1*, *Col3a1*, *Acta2*, *Tgfb1*, and *Ptgs2*. The expression of the COX‐2 protein, encoded by the *Ptgs2* gene, was also consistent with the increased messenger RNA (mRNA) expression of its encoding gene after ISP administration.^40^ A different study found an increased expression of cardiac genes, including *Tgfb1*, *Clo1a1*, *Ccl2*, and *Anp*, in ISP‐administered rats.^97^

Another pathway that is correlated with TGF‐β1‐induced cardiac fibrosis is the activation of the transient receptor potential melastatin 7 (TRPM7), a profibrotic mediator that stimulates the proliferation of fibroblasts and promotes the synthesis of collagen I. TRPM7 can be targeted, regulated, and inhibited by the microRNA, miR‐135a, by suppressing the translation or by inducing the degradation of the targeted gene, which in turn protects against ISP‐induced fibrosis through TRPM7 channel inhibition.^18^ In a model of ISP‐induced myocardial fibrosis, ISP was reported to increase the level of TRPM7 accompanied by a decrease in miR‐135a level. The same report indicated a positive feedback between TGF‐β1 and TRPM7, which was evident by the remarkable suppression of TGF‐β1 expression after the inactivation of the *TRPM7* gene.^64^

An additional mechanism observed to play a role in ISP‐induced cardiac fibrosis is the upregulation of microRNA‐214, which mediates proliferation, collagen synthesis, Mfn2 inhibition, and ERK1/2 MAPK signaling activation of fibroblasts (M.^118^). Because macrophages are present near myofibroblasts and contribute to heart injury in the early stages of heart insult preceding the fibrosis, the infiltration of macrophages was evaluated to determine the effect of ISP on macrophage activation. Continuous ISP infusion was also found to decrease microRNA (miR)‐133a levels. miR‐133a is ubiquitously expressed in the heart and is involved in orchestrating genetic reprogramming and modifying the protein makeup in response to injury.^50^ Therefore, to prevent genetic cardiac hypertrophy and decrease the expression of growth factors, complementary sequences on mRNA should be bound to suppress the translation of the corresponding proteins.^9^

ISP is also reported to cause significant loss in the left ventricle and replace with collagenous fiber, resulting in a proportional decrease in the area of viable myocytes. On the contrary, the same study reported ISP‐induced structural and functional changes in the heart. An echocardiography test showed an increase in the posterior wall thickness, interventricular septum wall thickness, and heart rate after ISP administration. In contrast, other cardiac parameters, including left ventricular end‐diastolic dimension, left ventricular end‐systolic dimension, ejection fraction, and fractional shortening, were lower in the ISP group compared to the control group.^8^ Additionally, ISP is capable of increasing the plasma level of biochemical indexes, including lactate dehydrogenase, aspartate aminotransferase, creatine kinase isoenzymes (CK‐MB), creatine kinase, and α‐hydroxybutyrate dehydrogenase, that are indicative of myocardial injury.^84^

#### 2.1.2 ISP induces oxidative stress

Oxidative stress mediates cardiac fibrogenesis, in which the exposure of fibroblasts to superoxide anions and other reactive species stimulates their differentiation, the release of TGF‐β, and the initiation of a fibrogenic response.^119^ Superoxide dismutase (SOD) is a scavenging enzyme capable of eliminating the generated reactive oxygen and nitrogen species and was found to exhibit decreased activity after the administration of ISP with a concomitant increase in malondialdehyde (L.^37^). Besides, ISP impairs the endogenous antioxidant defense and decreases antioxidant levels.^85^ The imbalance in antioxidant levels and oxidative stress was further reported after the administration of ISP, which was shown to cause a decrease in glutathione (GSH), catalase, and SOD and an increase in myocardial Thiobarbituric acid‐reactive substance (TBARS) level.^98^ This change in the oxidative indicators was correlated to increased nicotinamide adenine dinucleotide phosphate (NADPH) oxidase activity in ISP‐administered mice in a different study.^13^

More interestingly, cardiac fibrosis has been suggested to be linked to the loss of sirtuin 1 (SIRT1) expression. SIRT1 is a nicotinamide adenine dinucleotide (NAD+)–dependent histone deacetylase (HDAC) that functions as a transcription factor for various physiological processes through its downstream mediators PGC‐1α (proliferator‐activated receptor gamma coactivator 1‐alpha) and fibroblast growth factor 21.^120^ A reduction in SIRT1 expression leads to chronic and unopposed TGF‐β signaling through the activation of its receptor that causes the translocation of TGF‐β intermediates, namely Smad‐2 and Smad‐3, culminating in cardiac hypertrophy and fibrosis.^121^ ISP was shown to suppress the expression of SIRT1 at mRNA and protein levels, which in turn enhanced the transition of endothelial cells to mesenchymal cells and increased mesenchymal‐specific proteins, including α‐SMA and fibroblast‐specific protein 1 (FSP‐1).^33^ In a separate report, the gene expression of NADPH oxidase enzyme subunits, including *Cyba, Cybb, Ncf1, Ncf4,* and *Rac2* that participate in the production of oxidants, was shown to be induced remarkably by ISP infusion.^48^

Further, SIRT1 interacts with forkhead box O (FOXO) protein protecting against oxidative stress by upregulating the deacetylation of FOXO, promoting the FOXO/MnSOD (manganese superoxide dismutase) pathway, increasing the expression of MnSOD, and resisting oxidative stress.^122^ MnSOD is a reactive oxygen species (ROS)‐detoxifying enzyme regulated by FOXO3a, which transactivates MnSOD by binding to the gene promoter region of MnSOD leading to gene transcription promotion. On the contrary, FOXO3a is activated by SIRT1‐exerted deacetylation, increasing the expression of FOXO3a target genes and enhancing its stress resistance response.^123^ Decreased MnSOD has been shown to induce myocardial hypertrophy, and the restoration of its activity was demonstrated to protect against heart failure.^124^ ISO administration has been found to cause a downregulation in the protein expression of SIRT1, FOXO3a, and MnSOD, contributing to myocardial remodeling induction.^66^

Moreover, the defect in PPAR‐γ activity is reported to contribute to the pathological condition of fibrosis. It was demonstrated that mice with defective PPAR‐γ developed more severe cardiac fibrosis compared with the control group.^125^ In contrast, the activation and overexpression of PPAR‐γ are shown to be protective against the development of cardiac fibrosis through the inhibition of TGF‐β mediated accumulation of collagen.^126^ In line with these observations, ISP was observed to downregulate the expression of PPAR‐γ, which correlated with the developed fibrosis.^34^

#### 2.1.3 ISP induces proinflammatory mediators and perturbs immune‐inflammatory cascades

An inflammatory response is a physiological defense mechanism of the body against injurious stimuli. Active resolution is required for facilitating tissue recovery and healing. However, a failed resolution process leads to persistent inflammation, aggravated tissue destruction, and progressive fibrosis.^127^ Several mechanisms act as the interplay between inflammation and fibrosis. Persistent stimulation of adrenergic receptors causes cardiomyopathy through the release of proinflammatory mediators in the myocardium, which activates the downstream signaling pathway of NF‐κB. The expression of NF‐κB increased significantly in the ISP group compared to the control group. ISP also upregulated the expression of toll‐like receptor (TLR4), which would further participate in the elevated expression of NF‐κB.^42^

On the contrary, ISP‐induced cardiomyopathy triggered a remarkable increase in the proinflammatory cells (CD86+) concurrently with a reduction in the infiltration of anti‐inflammatory cells (CD206+). This was further validated by the upregulation of proinflammatory mediators, inducible nitric oxide synthase (iNOS), interleukin (IL‐1β) , IL‐6, and TNF‐α (tumor necrosis factor‐α), and the downregulation in the anti‐inflammatory markers, arginase‐1 and IL‐10. ISP also induced the proinflammatory responses by triggering the nuclear translocation of G‐protein‐coupled receptor kinase 5 and decreased the Cyclic adenosine monophosphate (cAMP) monocyte chemotactic protein‐1 concentration hindering its mediated immunoregulatory effect.^36^

ISP caused an increase in the infiltration of CD45 and CD68 cells, which was evident by the higher number of immunolabeled cells compared with the control group. The ISP‐stimulated macrophages triggered the myofibroblasts to release fibrotic markers that further aggravated the fibrotic injury. The CCR2 receptor levels were also elevated in response to ISP administration. Macrophages play a pivotal role in ECM remodeling and the secretion of ECM components. They are the primary cells responsible for pathogen phagocytosis, which in turn can alter the phenotypic features of macrophages. For instance, the engulfment of apoptotic neutrophils and the release of cytokines induce the conversion to the profibrotic phenotype (M2) of the naive (M0) macrophages.^127^ It is known that depending on CCR2 signaling, macrophages increasingly migrate and infiltrate cardiac tissue in response to heart injury. This is observed by the significantly higher infiltration of M1 and M2 macrophages in the myocardium of ISP‐administered rats.^69^ In this regard, it is reported that β‐adrenergic activation causes macrophage infiltration within the myocardium and a subsequent cardiac remodeling represented as fibrosis (H.^20^). The profibrotic role of macrophages was more elaborately studied, showing the tendency of macrophages to adopt various immunophenotypes during different phases of fibrosis induction. CD68^+^ M1 macrophages were realized early on in the inflammatory phase ranging from days 1 to 7 after the initiation of the inflammatory response. Thereafter, CD163+ M2 was observed to start infiltrating the injured tissue during the reparative phase from days 7 to 28. Other types such as CD204‐ and MHC class II‐positive macrophages were also detected in response to ISP‐induced myocardial fibrosis.^21^

Persistent immune response after myocardial injury is thought to lead to detrimental consequences. Regulatory T (Treg) cells orchestrate immune homeostasis and suppress the activation of effector T cells, which are pathologically activated causing tissue cardiac remodeling. The forkhead/winged helix transcription factor (Fox) p3 is an essential marker for the development of Treg cells, and its production is necessary for the maintenance of functional Treg cells.^128^ Foxp3 suppresses the myocardial infiltration of inflammatory immune cells and therefore ameliorates cardiac hypertrophy and remodeling.^129^ The myocardial expression of Foxp3 was markedly decreased in ISP‐induced hypertrophy estimated by protein levels using immunohistochemistry and mRNA levels using polymerase chain reaction.^38^

Galectin‐3 is one of the galactoside‐binding lectins, secreted by macrophages under the influence of different mediators and expressed fibroblasts, and endothelial and other cell types. It is involved in mediating cardiac fibrosis through its wide range of cellular functions. It promotes cellular differentiation, causing the activation of cardiac fibroblasts, which leads to the release of matrix proteins. It also contributes to inflammatory response and modulates the immune response in the heart by elevating the level of proinflammatory cytokines.^130^ A study has confirmed that the enhanced expression observed after ISP infusion correlated with marked inflammation and fibrogenesis.^7^

Furthermore, microsomal prostaglandin E synthase‐1 (mPGES‐1) is a dominant source for PGE2 implicated in inflammation, pain, and aggravated myocardial injury. In contrast, the deletion of mPGES‐1 is proved to be therapeutically beneficial in attenuating vascular injury. ISP was observed to cause an elevation in the mPGES‐1 level, which associated with an augmentation in the resulting cardiac fibrosis. However, the deletion of mPGES‐1 by small interfering RNA subdued the induced fibrosis after ISP administration in mPGES‐1 knockout mice.^16^

In a different approach, ISP was demonstrated to block the signal transducer and activator of transcription 6 (STAT6), which regulates and maintains a balance in immune cell activation in response to inflammatory response.^131^ This blockage led to an aggravated immune response represented by an enhanced infiltration of CD11b + myeloid cells and differentiation of CD11b^+^Ly6C^+/low^ macrophages accompanied by a release of IL‐1α, IL‐18, and TGF‐β.^23^

#### 2.1.4 ISP dysregulates calcium homeostasis

Calcium (Ca^2+^) is inextricably linked to cardiac function as well as dysfunction under pathological conditions. Ca^2+^ is essential for stimulating the energy production from the mitochondria required for heart contraction. It serves as a signal to activate the mitochondrial hydrogenases and ATP synthase for ATP synthesis. However, cytosolic Ca^2+^ overload triggers the mitochondrial death pathway by initiating the mitochondrial permeability transition pore, permeabilization, and dysfunction.^132^ The mitochondrial impairment induced by ISP was also evident in a study that demonstrated a decrease in the oxygen consumption and respiratory control index after ISP injection. It also showed the induced reduction in the subunits of mitochondrial respiratory chain complexes as follows: complex V alpha (CV‐ATP5A), complex III core protein 2 (cytochrome b‐c1 complex subunit 2, CIII‐UQCRC2), complex IV subunit I (cytochrome c oxidase I, CIV‐MTCO1), complex II subunit 30 (succinate dehydrogenase [ubiquinone] iron–sulfur subunit, CII‐SDHB), and complex I subunit NDUF8 (NADH dehydrogenase [ubiquinone] 1 beta subcomplex subunit 8, CI‐NDUFB8).^73^

Several mechanisms are responsible for regulating the Ca^2+^ concentration and maintaining its homeostasis. An interesting mechanism reported to play a role in ISP‐induced cardiac fibrosis is through mediating Ca^2+^ dysregulation after induction of phospholamban expression, which aggravates cardiac fibrosis and hypertrophy.^133^ Phospholamban is a reversible inhibitor of the sarcoplasmic reticulum Ca^2+^‐ATPase (SERCA2A), which regulates Ca^2+^ sequestration in the sarcoplasmic reticulum of the myocyte and in turn maintains excitation–contraction coupling of the myocytes and synchronized cardiac contractility.^134^ Evidence showed that altered phospholamban levels and the subsequent alteration in Ca^2+^ uptake and release by the sarcoplasmic reticulum are indicated in failing hearts as well as myocardial remodeling,^135^ and the ablation of phospholamban is protective against cardiac dysfunction.^136^

**FIGURE 2:**
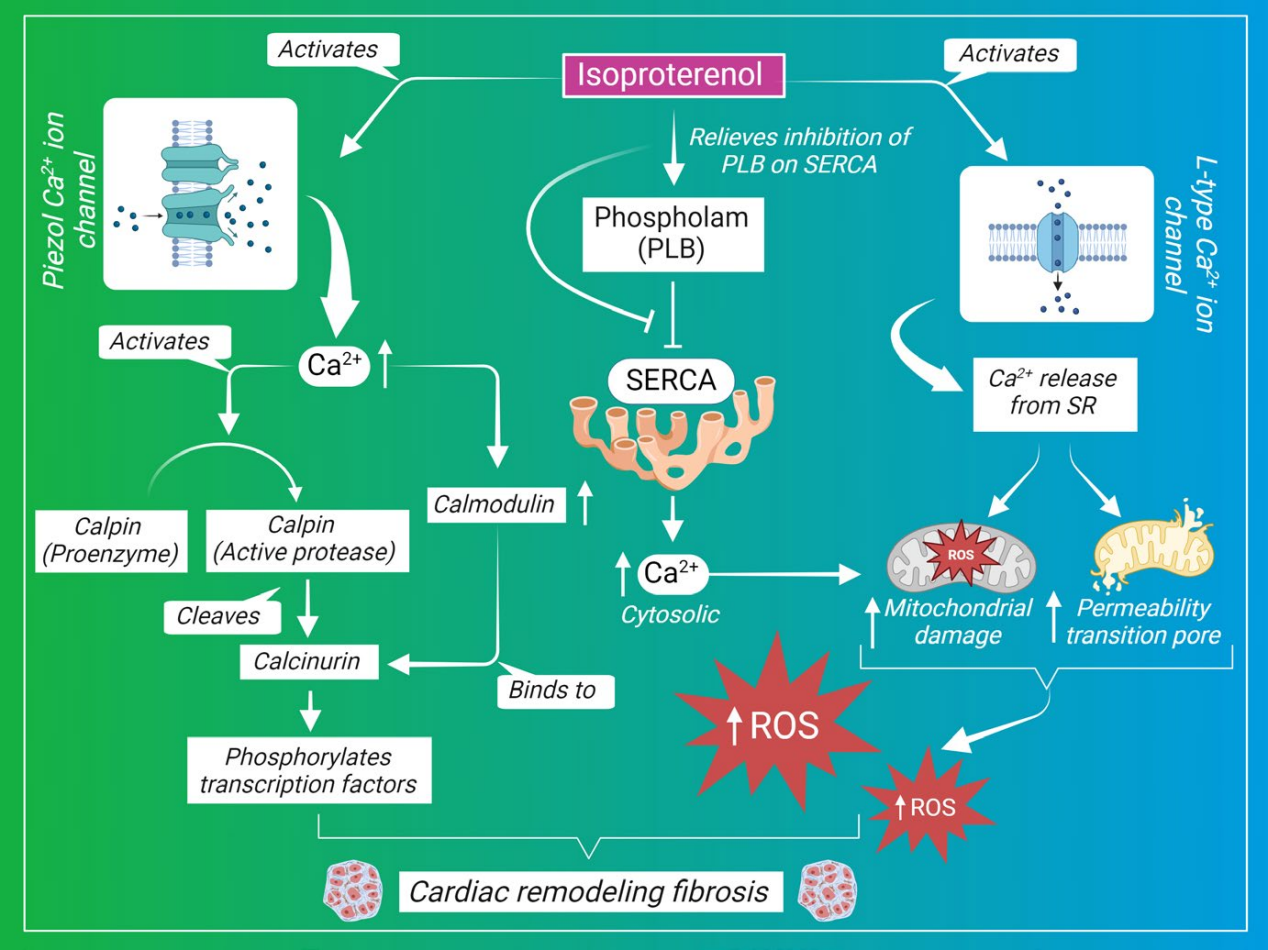
Ca^2+^‐dependent mechanisms of ISP (isoproterenol)–induced cardiac fibrosis.

Similarly, the impact of calcium homeostasis impairment on pathological fibrosis was investigated by assessing the role of the Piezo1‐permeable Ca^2+^ ion channel in maintaining regulated Ca^2+^ concentration. Piezo1 is a mechanosensitive ion channel that regulates Ca^2+^ under hypertrophic stimuli in cardiomyocytes. It is usually upregulated in human cardiac diseases in response to autonomically sensed stresses and induced cardiac hypertrophy by activating the Ca^2+^ hypertrophy pathway represented by calcineurin and calpain. ISP has led to an elevated Ca^2+^ transient amplitude, which was suppressed by Piezo1 knockout. The neurohumoral stimulation by ISP enhanced heart contractility and the stretch‐related ion channel activation of Piezo1, which in turn elevated Ca^2+^ influx. This elucidates that Piezo1 is involved in ISP‐induced Ca^2^ elevation.^137^ Mechanisms of ISP that are Ca^2+^ dependent are shown in Figure 2.

Another mechanism involved in dysregulating calcium homeostasis and aggravating the hypertrophic response is the effect of ISP on Ca^2+^ intracellular concentration and current. ISP was shown to increase the peak value of l‐type Ca^2+^ current (*I*_Ca_) and to enhance the Ca^2+^ transient amplitude determined by measuring Ca^2+^ current through l‐type Ca^2+^ channels, which stimulates further release from sarcoplasmic reticulum. The increase in [Ca^2+^]_I_ (the Ca^2+^ transient amplitude) is a key factor in the development of cardiac hypertrophy due to its role in maintaining excitation–contraction coupling.^99^ As intrinsically Ca^2+^ is a housekeeper mediator of homeostatic cardiac electrical conductivity and contraction, in a different approach ISP was shown to induce ventricular arrhythmias, decrease the duration of the action potential from the peak to repolarization, and disrupt the action potential and Ca^2+^ signal coupling with electrical stimulation.^138^ Collectively, altered Ca^2+^ is believed to be one of the major regulators of ISP‐induced fibrosis and hypertrophy, and it represents an important therapeutic target for further exploration of therapeutics.

#### 2.1.5 ISP induces autophagy

Autophagy is a catabolic process in which long‐lived proteins or other dysfunctional cellular components are degraded by this lysosomal degradation pathway. It also participates in regulating the hypertrophic response of the heart, where hypertrophy leading stressors can induce autophagy in the heart (Liu et al., 2018).^139^ Autophagy imbalance is thought to be linked with cardiac fibrosis when the heart moves from the early compensatory phase to the late decomposition phase.^6^ For instance, the aortic constriction–induced cardiac hypertrophy has been shown to induce an increase in the autophagy markers, including LC3‐II, Atg5, and beclin‐1.^140^

Mammalian target of rapamycin (mTOR) signaling activation exerts a cardioprotective effect by inhibiting autophagy. ISP has been demonstrated to decrease the mRNA level of mTOR as well as the expression of the phosphorylated mTOR. In contrast, the mRNA expression of autophagy markers LC3‐II and beclin‐1 increased after ISP administration. α‐Myosin heavy chain (α‐MHC), β‐MHC, atrial natriuretic peptide (ANP), and brain natriuretic peptide (BNP), the main hypertrophic markers known to be associated with cardiac hypertrophy and fibrosis, were significantly induced by ISP treatment.^63^, ^141^ Autophagy is known to be regulated by two main modulators: mTOR and AMPK (adenosine 5′‐monophosphate [AMP]‐activated protein kinase). mTOR acts as a metabolic sensor to negatively regulate autophagy through PI3K/Akt/MAPK signaling, whereas AMPK inhibits mTOR and phosphorylates Unc‐51‐like autophagy‐activating kinase 1 (ULK1), which in turn forms an isolation membrane, fundamental for the formation of the autophagosome.^142^ It has been reported that the administration of ISP caused AMPK signaling suppression, mTOR activation, and an upregulation in the phosphorylation of ULK1 757 locus resulting in decreased autophagy, the protective machinery against the induced cardiac fibrosis and hypertrophy.^93^ To confirm this, a recent study has implicated the impact of ISP on perturbing the autophagic flux by stimulating the release of LC3‐II and P62 and the accumulation of the autophagosome and by altering the balance of the mitochondrial fission and fusion by elevating the corresponding proteins possibly by decreasing SIRT1 expression as well as the activity of transcription factor EB.^22^

As the role of autophagy in every disorder remains controversial, a recent study has also revealed some disparity in the exact role of ISP related to autophagy. An aberrant autophagy induction was observed after the stimulation of neonatal rat cardiomyocytes with ISP. This was apparent by the evaluated autophagic markers that were significantly reduced after ISP stimulation. ISP has led to reduced autophagic flux, beclin‐1, Atg5 expression, and LC3‐II/I ratio, in addition to downregulated phosphorylation of mTOR.

#### 2.1.6 ISP alters gap junction structure

Gap junctions are intracellular structures that aid in cellular communication and the passage of ionic and other molecules between cells. They also mediate electrical coupling, spreading the electrical excitation in an ordered and organized manner. The gap junction consists of a hemichannel and a transmembrane protein called connexins. Connexins 43 (Cx43) is most abundant in heart myocytes and is normally distributed over the intercalated disc. However, in heart diseases such as hypertrophy, cardiomyopathy was associated with a defective distribution pattern of Cx43, which was observed to be localized at the lateral side of the myocyte membrane.^143^ The decreased Cx43 expression was correlated with increased collagen under various pathological conditions, resulting in impaired conduction of cardiac impulse.^144^

In the ISP‐administered heart, immunolabeling of Cx43 detected an enhanced displacement of the electron coupling protein Cx43 gap junctions to the lateral side of cardiomyocytes and a conventional polar localization at the intercalated disc, which was accompanied with diffuse collagen deposition. The hypertrophied hearts also showed an increase in the phosphorylation of Cx43 at serine 368 and disordered topology modulating cardiac remodeling.^96^ The same effect of ISP on the displacement, redistribution, and decreased expression of Cx43 is also reported in another study. The study indicated the possible involvement of ATP‐sensitive K+ (KATP) in ISP‐induced effect, where it would antagonize or inhibit the desired action of the channel. However, the administration of KATP channel agonist nicorandil attenuated the resulting hypertrophy induced by ISP.^24^

#### 2.1.7 ISP induces the activity of angiotensin‐converting enzymes

The renin–angiotensin system (RAS) is a humoral system that regulates blood pressure and salt homeostasis. The main player in this system is the angiotensin‐converting enzyme (ACE) that converts angiotensin I into angiotensin II. The activation of RAS components, including ACE, and the subsequent production of angiotensin II are proposed to induce the development of cardiac remodeling because angiotensin II is known to play a pathological role in promoting fibroblast differentiation and hyperplasia.

The ACE gene expression is regulated by growth factors, steroid hormones, and β‐adrenergic agonists.^145^ An experimental study has demonstrated that the ACE activities of the left and right ventricles increased significantly by 2.7‐ and 1.9‐folds 1 day after ISP administration. The increase in ACE activity correlated with increased ACE mRNA expression, which was evident by the densitometric analysis of ACE complementary DNA in reverse transcription‐polymerase chain reaction showing a 1.9‐fold increase in mRNA in the left ventricle.^10^

### 2.2 Molecular mechanisms

#### 2.2.1 ISP increases the activity of HDACs

HDACs are enzymes that maintain balanced acetylated/deacetylated states of histones along with one acetyltransferase. Acetylation of histones provides a relaxed histone structure that is needed for regulating the transcription activation; however, HDACs remove the acetyl groups hindering gene transcription. Other HDACs are not capable of deacetylating histones due to their mutated catalytic domain, which instead have the N‐terminal domain that allows their interaction with transcription factors and transcription suppressors.^146^ The connection between HDACs and cardiac remodeling is attributed to the discovered interaction between HDACs and myocyte enhancer factor‐2 transcription factors, which are the main regulators of cardiac hypertrophy.^147^

HDACs are categorized into four classes: I, II, III, and IV. Class I HDACs (HDAC1, HDAC2, HDAC3, and HDAC8) are considered as pro‐hypertrophic factors and opposing regulators of cardiac hypertrophy, where their inhibition confers protection against the incited hypertrophic response.^148^ Heart overexpression of HDAC2 is linked with reduced lysine 27 in histone 3 acetylation causing an enhanced transcriptional activity.^149^ Heart exposure to a variety of hypertrophic stimuli predisposes the expression of fetal genes associated with myocyte hypertrophy. The role of histone deacetylase‐2 (HDAC2) is documented for its involvement in stimulating the expression of fetal cardiac isoforms and augmenting the hypertrophic response through the inactivation of glycogen synthase kinase‐3β (Gsk3β). It was also shown that HDAC2‐deficient mice are resistant to cardiac remodeling induced by ISP infusion.^150^ An elevated expression of p‐HDAC2, and its downstream proteins p‐AKT and p‐GSK3β, was found in ISP‐induced cardiac hypertrophy in both in vitro and in vivo models.^31^ Other histone‐modifying enzymes have been reported for their role in cardiac remodeling. Set7 is a methyltransferase enzyme that participates in chromatin remodeling and targeting lysine residues on histones by methylation, leading to epigenetic changes in chromatin and posttranslational modifications implicated in fibrotic response to chronic stresses.^151^ The knockout of Set7 in mice injected with ISP resulted in an attenuation in myocardial fibrosis, showing the role of Set7 played in mediating cardiac remodeling in response to cardiac cellular insult or stress.

Peroxisome PGC‐1α; in the context of transcription regulation, the signal transducer and activator of transcription 3 (STAT3), an important transcription factor, plays a protective role in the heart through its genomic activities by upregulating antioxidative and antiapoptotic genes in cardiomyocytes.^152^ However, the activation of STAT3 promotes cellular survival and proliferation as well as collagen biosynthesis in fibroblasts.^153^ In an ISP‐induced cardiac fibrosis, the administration of C188‐9 has significantly alleviated the development of myocardial fibrosis. C188‐9 is a synthetic small molecule that inhibits the phosphorylation and activation of STAT3 by targeting the Src homology 2 (SH2) domain. C188‐9 decreased the level of Col1a1, Col1a2, Col3a1, and α‐SMA expression and downregulated the phosphorylated form of STAT3, suggesting STAT3 as an intracellular target of ISP in inducing fibrosis.^105^

Similarly, nuclear receptor Nur77 is one of the NR4A nuclear receptors that has emerged as a regulator neurohormonal mechanism implicated in cardiac response to stress. NR4A acts as NGFI‐B‐ or Nur‐response elements (NBRE or NurRE) found in the promoter region of gene sequence.^154^ As reported previously, Nur77 attenuates adverse cardiac remodeling induced after myocardium infarction.^155^ In contrast, Nur77 knockout is reported to augment cardiac fibrosis after myocardial infarction, where the deficiency of Nur77 enhanced endothelial‐to‐mesenchymal transition (EndMT) that is evident by the increased expression of FSP‐1, SM22α, and Snail (EndMT) and the decreased expression of Platelet endothelial cell adhesion molecule‐1 (PECAM‐1) (endothelial marker) and Endothelial nitric oxide synthase (eNOS).^156^ In a model of ISP‐induced cardiac fibrosis, ISP was found to cause severe thinning and rupture in the myocardial tissue and transformation of fibroblast into myofibroblast phenotype in Nur77 knockout mice. However, the presence of normally functioning Nur77 conferred protection against ISP‐induced effects.^157^ Obviously, this has been studied in depth, as recently it was found that ISP upregulates the enhancer of zeste homolog 2 (EZH2), which is a catalytic subunit part of the polycomb repressive complex 2 that can affect and regulate the activity of its target genes through trimethylating lysine 27 on histone 3 (H3K27me3) (T.^158^). The report revealed that both EZH2 and H3K27me3 were overexpressed after ISP treatment.^159^

#### 2.2.2 ISP increases the expression of poly(ADP‐ribose)polymerase 1

Poly(ADP‐ribose)polymerase 1 (PARP‐1) is a nuclear enzyme that is documented to mediate myocardial hypertrophy and cardiac remodeling. Multiple lines of evidence support the fact that reactive oxygen and nitrogen species are produced during various types of cardiomyopathies as a product of different sources. Extensive oxidative and nitrosative stress triggers extreme damage to DNA, overactivation of PARP‐1, subsequent depletion of PARP‐1 substrate stores, NAD^+^ and mitochondrial electron chain impairment, ATP depletion, and eventually cellular dysfunction and death. Therefore, the inhibition of PARP‐1 is considered as a protective mechanism that would reverse the aforementioned defective consequences.^160^ The therapeutic potential of PARP‐1 has been experimentally performed, where the transfection of cultured cardiac fibroblasts with small interference RNA‐PARP‐1 suppressing the activity of PARP‐1 led to inhibition of TGF‐β‐induced proliferation and differentiation of fibroblasts, and the pharmacological inhibition of PARP‐1 by the administration of PARP‐1 inhibitor; 4‐aminobenzamide abrogated the induced cardiac fibrosis. This together demonstrates that there is a vital connection between PARP‐1 activation and fibrosis induction.^161^ Consistently, ISP induced cardiac remodeling by increasing the expression of PARP‐1 and its activity, PARylation, which triggered subsequent cell injury.^19^

#### 2.2.3 ISP induces the expression of heat shock proteins

Heat shock proteins (HSP) are stress proteins that are expressed in response to various physiological insults. Heat shock factor 1 (HSF‐1) is the main regulating factor for the expression of HSPs and their genes. HSPs help preserve protein quality through maintaining balanced protein folding, synthesis, and turnover. Besides, they are collagen‐specific molecular proteins, where their expression triggers collagen production, and therefore, it can be said that they enhance fibrosis progression.^162^ A study has suggested that ISP‐mediated increase in collagen expression is dependent on the transcriptional effects of HSF‐1. They demonstrated that ISP treatment significantly increased the protein expression of HSF‐1 in the nuclei of cardiac cells, concomitantly with an elevated activity through the detected upregulated phosphorylated HSF‐1 level. ISP also induced HSP47 expression, which is closely correlated to fibrosis.^11^

#### 2.2.4 ISP upregulates the expression of urotensin II peptides

The urotensinergic system plays a physiological role in regulating the hemodynamic functions of the heart. This peptidergic system through its urotensin II peptide exerts different biological effects, including positive inotropic and chronotropic responses, in addition to the induction of collagen and fibronectin accumulation.^163^ Therefore, it is thought that it is linked to cardiac pathophysiological conditions, including hypertrophy, hypertension, and heart failure, due to promitogenic and hypertrophic effects on cardiomyocytes by the action of urotensin II on its receptor. Urotensin is a somatostatin‐like peptide and a vasoconstrictor cyclic peptide that potently stimulates smooth muscles and is considered the endogenous ligand of the G‐protein‐coupled receptor.^164^

The ventricular content of urotensin II was reported to be increased after the administration of ISP, which is thought to be involved in mediating the cardiac fibrogenesis of ISP, which in turn potentiated the mRNA expression of urotensin II receptor, angiotensin II, and promoted collagen synthesis.^15^ Postmyocardial infarction was also previously associated with a significant increase of 75% in urotensin II peptide and urotensin II receptor protein expression with a concomitant increase in the mRNA transcripts in procollagen and fibronectin.^165^ The critical role of urotensin II was recently confirmed in cardiac fibrosis by modulating TGF‐β/Smad and activating the fibrotic response.^166^ This indicates that urotensin II is a determinant in cardiac pathological conditions characterized by increased urotensin II receptors.

#### 2.2.5 ISP augments thioredoxin domain containing 5

Endoplasmic reticulum is responsible for correct protein folding, which is accomplished by the protein disulfide isomerase protein family. Thioredoxin domain containing 5 (TXNDC5) is a member of this family, assisting in folding of newly synthesized proteins into their mature form. It also facilitates recycling damaged molecules and long‐lived proteins through disulfide bond exchange reactions performed by thioredoxin domain.^167^ TXNDC5 is a cardiac fibroblast–enriched endoplasmic reticulum protein that promotes ECM protein production and folding and cardiac fibrosis through redox‐mediated response by enhancing theJun N‐terminal kinase activity; therefore, it is considered as a novel mediator of cardiac fibrosis. A dysregulated expression of TXNDC5 is linked with oxidative stress, cellular aging, and various pathological conditions such as cancer, diabetes, and neurodegenerative diseases.^168^

ISP was shown to induce cardiac fibrosis by upregulating TXNDC5, which is positively correlated with the expression of transforming growth factor beta 1 (TGF‐β1).^14^ The mRNA transcript expression of TXNDC5 was found upregulated in patients with atrial fibrosis, with a positive correlation with transcripts encoding TGF‐β1 and ECM proteins. This overexpression triggered the activation and proliferation of atrial fibroblasts and ECM protein production. In contrast, the knockdown of TXNDC5 significantly attenuated the induced fibrosis. In addition, transgenic mice that constitutively overexpress active TGF‐β exhibited a strong concomitant expression of TXNDC5 along with collagen deposition.^169^ This reveals that the endoplasmic reticulum protein TXNDC5 aggravates cardiac fibrosis, and its targeted deletion protects against it.

#### 2.2.6 ISP downregulates phosphatase and tensin homolog protein

Phosphatase and tensin homolog (PTEN) protein is a dual‐specific protein tyrosine phosphatase known as a tumor suppressor gene that controls and restricts tumor development. PTEN dephosphorylates phosphatidylinositol to produce phosphatidylinositol 4,5‐bisphosphate and negatively regulates PI3K/Akt signaling pathway. A convincing number of studies demonstrated the participation of PTEN in the progression of myocardial infarction and the subsequent associated cardiac remodeling.^170^, ^171^ PTEN expression associated with myocardial infarction induction was shown to deteriorate cardiac remodeling through inhibiting PI3K/Akt signaling pathway.^172^ However, the case with ISP was found to be contrary to what has been previously shown.

ISP has been demonstrated to induce cardiac hypertrophy, and fibrosis is attributed to the involvement of PTEN/AKT/mTOR pathway, a downstream signaling pathway known for its role in pathological conditions, including cardiac hypertrophy and fibrosis, where the downregulation of PTEN and the phosphorylation of AKT/mTOR play a role in the pathogenesis of cardiac hypertrophy and fibrosis and the reversal of which would act as a protection against both cardiac hypertrophy and fibrosis.^32^ This was further confirmed by a study that linked the ISP‐induced cardiac hypertrophy with PTEN pathway. Moreover, ISP was observed to upregulate the expression of liver kinase B1 interacting protein 1 (LKB1IP), which positively promotes ISP‐induced cardiac hypertrophy through activating AKT signaling by directly targeting and interacting with PTEN (a negative regulator of AKT phosphorylation) and inhibiting its phosphatase activity. The interaction between LKB1IP and PTEN increased after ISP treatment.^12^

#### 2.2.7 ISP induces the expression of RNA‐binding protein muscleblind‐like1 gene

At the genetic level, RNA‐binding protein muscleblind‐like1 (MBNL1) determines the cell's transcriptional state through modulating alternative splicing, polyadenylation, and mRNA localization by binding to selective transcripts, and promotes the differentiation of fibroblasts to myofibroblasts mediating fibrotic remodeling.^173^ The overexpression of MBNL1 in cardiac fibroblasts enhanced their transition to myofibroblasts through transcriptome maturation.^174^

MBNL1 activates the nodal signaling axes and interacts with the RNA machinery processes, encoding for differentiation‐specific signaling that promotes fibroblast differentiation and altering their proteome into myofibroblast‐based structure and function and augmenting fibrotic remodeling.^175^ A notable increase in fibrosis induced by ISP was accompanied by an overexpression in MBNL1 protein. MBNL1 has been demonstrated to exhibit ISP‐induced myocardial remodeling by inducing the expression of the myocardial protein, which in turn is considered as an important inducer of myocardial hypertrophy.^176^

## 3 MEDICINAL PLANTS EXHIBITED THERAPEUTIC AND PREVENTIVE POTENTIAL IN ISP‐INDUCED CARDIAC FIBROSIS

Plants have been used in medicines since time immemorial for their therapeutic and preventive benefits. Plants, including vegetables and spices, have been well utilized for their nutritional benefits and have demonstrated beneficial effects in numerous chronic diseases in which oxidative stress, inflammation, and apoptosis play significant roles. Plants as medicine for cardiovascular drugs are best represented by the use of digoxin in cardiogenic shock, which is obtained from *Digitalis purpurea*, popularly known as foxglove. Plants effective against ISP‐induced myocardial fibrosis and their mechanism are summarized in Table 4. Many plant extracts, including *Cymbopogon proximus*, *Terminalia arjuna* (Roxb.), *Dendrobium candidum*, *Panax notoginseng*, Danshen formulae, *Gentianella acuta*, blueberry, and citrus pectin, have been evaluated in ISP‐induced myocardial fibrosis. Many of them have been evaluated in rat models of ISP‐induced myocardial fibrosis. The dose regimen chosen for ISP was 5 mg/kg, s.c. (subcutaneous) for 7–14 days, and the test agent was administered for 7 days prior to as well as along with the ISP dose to demonstrate the preventive as well as therapeutic effects.

Many formulations containing plant extracts and traditional Chinese medicines, including Huoxin Pill and Dangshen Erling and Si‐Miao‐Yong decoction, have been evaluated in ISP‐induced myocardial fibrosis and shown to be effective in reducing oxidative stress, inflammation, apoptosis, and fibrosis. The available data mostly from experimental models reveal the potential of the plants in alleviating cardiac remodeling, as evidenced by their effect on the expression of fibrotic proteins and the activation of related signaling pathways that lead to the induction of fibrotic markers. A few of them also demonstrated the effect of the plant extract on the functional parameters of the heart.^84^ However, most other studies have investigated the impact of herbal extracts or formulations only on major known signaling cascades that are predominantly related to fibrosis induction. This comprehensive review shows that there are other new targets that are rarely investigated in fibrosis research, yet they are prominently implicated in cardiac fibrosis pathogenesis. Therefore, it is important to identify the constituents of medicinal herbs for their beneficial effects. Identification of the active constituents in plant extracts will facilitate future drug discovery and development. Whole‐herb extracts can be well utilized for nutraceutical purposes, as traditional medicine always propagates the use of whole‐herb extracts and formulations based on the principles of synergy. Further, toxicological studies are needed for the evaluation of those medicinal plants in terms of side effects and tolerance so they can be extrapolated for human usage. There is a lack of modern medicines for treating cardiac fibrosis. Thus, herbal medicines, including single‐herb extracts, or polyherbal medicine should be evaluated for their cardioprotective effects in a comprehensive manner. Phytoconstituents need to be isolated, and their pharmacokinetics and toxicity must be determined to develop them as drugs for cardiac fibrosis.

## 4 PHYTOCHEMICALS EXHIBITED THERAPEUTIC AND PREVENTIVE POTENTIAL IN ISP‐INDUCED CARDIAC FIBROSIS

Naturally derived compounds are considered to have potential as antifibrotic treatments for various cardiologic diseases. Obviously, they exert anti‐inflammatory, antioxidative, antiproliferative effects and differentiation on cardiac fibroblasts. Although most of them are still in the preclinical stage and are being experimentally tested, in vitro and in vivo data reveal that they have shown promising potential in treating cardiologic diseases. As summarized in Tables 2 and 3, some phytochemicals, such as Astragaloside *and* Astaxanthin, have been extensively evaluated by multiple studies, illustrating their protective effect on improving the antioxidant effect and scavenging free radicals that impose oxidative damage on cardiomyocytes. Other implicated mechanisms include inhibiting the activation of transcription factors and enhancing the function of different microRNAs, which in turn inhibit the expression of fibrotic marker genes. Other phytochemicals demonstrated beneficial effects through the modulation of various fibrotic biomarkers such as hydroxyproline, collagen I, MMP‐9, TIMPs, NF‐κB, vascular endothelial growth factor (VEGF) Left ventricular end‐diastolic pressure , and platelet derived groth factor (PDGF), as has been previously suggested in a detailed report.^177^ However, it still requires to be additionally investigated at the molecular level to decipher the underlying intracellular signal transduction pathways in mediating the downregulation of fibrotic markers besides their impact on cardiac functional parameters, which is lacking in most of those experimentally assessed compounds so that they can be closely correlated with clinical application.

**TABLE 3:** Phytochemicals exhibited therapeutic potential in ISP‐induced myocardial remodeling and fibrosis in rat models.

Phytochemical (plant)	Compound dosing	ISP dosing regimen and species	Protective mechanisms	Reference
Curcumin (*Curcuma longa*)	200 mg/kg/day, intragastric for 4 weeks	5 mg/kg/day s.c. for 7 days to SD rats	↑mTOR ↓α‐MHC, β‐MHC, ANP, BNP, ↑LC3‐II and beclin‐1	[63]
Astragaloside IV (*Astragalus membranaceus*)	10 mg/kg/day orally on day 6 of modeling	5 mg/kg/day, s.c. for 14 days to SD rats	↑miR‐135a ↓TRPM7, ↓collagen I, ↓fibroblast proliferation ↓α‐SMA, TGF‐β/Smad	[64]
Farnesol	50 μM, i.p. for 8 days	4.5 mg/kg, i.p. for 8 days to Wistar albino rats	↓HW/BW, LVED, QRS complex duration, QTc interval and T wave ↓Fibrotic area, inflammatory cells, ROS ↑CAT and SOD ↑pAKT/AKT ↓Bax/Bcl‐2, pERK1/2/ERK1/2	[65]
Echinacoside (*Cistanches Herba*)	Rats: 20 μg/g i.p. 30 min before ISP for 2 weeks, cells: pretreated with 50 μM for 30 min prior to ISP	10 mg/kg/day i.p. for 2 weeks to SD rats 10 μM ISP to AC16 cells for 24 h	↓ROS, 8‐OHdG, HW/BW ↓carbonyl protein ↑LVEF, LVFS ↓LVIDd, LVIDs, IVSTd ↓Collagen ↑ SIRT1, FOXO3a, and MnSOD	[66]
Astragaloside	40 mg/kg/day for 30 days	10 mg/kg/d i.p. to SD rats for 30 days	↓TGF‐β1, Smad 2/3 and 4, Smad 7 ↓Collagen	[67]
Galangin (*Alpinia galangal*)	1 mg/kg, p.o. for 14 days	5 mg/kg s.c. for 14 days to albino Wistar rats	↓AST, ALT, LDH, CK, CK‐MB and the levels of cTnT and cTnI ↓TBARS, LHP ↓TNF‐α, IL‐6, NF‐ҡB, COX‐2, and iNOS, MMP‐2, MMP‐9, TGF‐β1, fibronectin	[68]
Berberine	10, 30, and 60 mg/kg, p.o. for 14 days	5 mg/kg, s.c. for 10 days to SD rats	↓CD45, CD68, CCR2, M1, and M2 macrophages	[69]
Quercetin	25 and 50 mg/kg	15 mg/kg s.c. for 21 days to Wistar rats	↓CTGF, TGF‐β1, collagen I, collagen III, fibronectin	[70]
Ginsenoside‐Re	5 or 20 mg/kg, p.o. for 4 weeks	5 mg/kg s.c. for 7 days to Wistar rats	↓HW, LVEDP, ↑LVSP ↓Collagen fibers, hydroxyproline ↓TGF‐β1, p‐Smad3	[71]
Astaxanthin	25 mg/kg, p.o. every day for 2 weeks	50 mg/kg s.c. twice a week for 2 weeks to rats	↓AST, ALT, ALP, and MDA ↑Catalase, SOD, GSH	[72]
Astaxanthin	150 mg/kg, p.o. for 2 weeks	85 mg/kg twice at an interval of 24 h at the end of the 2 weeks to Wistar rats	↑O_2_ consumption, ↑complex subunits, ↑ANT, CyP‐D	[73]
Astaxanthin	150 mg/kg p.o. for 4 weeks	100 mg/kg, s.c. twice with an interval of 24 h to Wistar rats	↑CV, CIII, CIV, CII, and CI subunits ↓H_2_O_2_, superoxide anion ↑SOD2, cardiolipin	[74]
Scutellarin	10 mg/kg and 20 mg/kg, i.p. for 15 days	5 mg/kg, s.c. for 7 days to SD rats	↑Microvascular density ↑CD31, ↑Jagged1, Notch 1, and Hes1	[75]
Icariin	10, 20, and 40 mg/kg, p.o. for 8 weeks	170 mg/kg, s.c. twice (separated by a 24‐h interval) to SD rats	↓MMP‐2, MMP‐9 ↓Bax, caspase 3 ↑Bcl‐2	[76]
Stachydrine	10 and 40 mg/kg, p.o. for 21 days	5 mg/kg, i.p. for 21 days to SD rats	↓IL‐6, TNF‐α, IFN‐γ and IFN‐1β, GSH, SOD, MDA, p‐STAT3 and p‐JAK2	[77]
Proanthocyanidins	50, 100, and 150 mg/kg, p.o. for 1 week	5 mg/kg, s.c. for 1 week to Wistar rats	↓MDA, ASK‐1, NF‐κB, COX‐2 ↑SOD	[78]
Leonurine	25, 50, and 100 mg/kg, p.o. for 48 days	5 mg/kg, s.c. on day 1, 2.5 mg/kg for 47 days to SD rats	↓GSDMD, caspase 1, IL‐1β	[79]
Shengmaiyin	3.26 and 13.04 g/kg, p.o. for 7 days	20, 10, and 5 mg/kg, s.c. for 3 days followed by 3 mg/kg for 4 days to SD rats	↓HIF1α, ADCY1, PKA, PPARα, and CPT1A	[80]
Aloin	25 and 50 mg/kg, p.o. for 14 days	5 mg/kg/day, s.c. for 14 days to SD rats	↓TGF‐β, pSmad2/3 ↑Nrf2, HO‐1	[81]
Isosteviol sodium	4 mg/kg for 7 days and 5 μmol/L	5 mg/kg, s.c. for 7 days to SD rats and 10 μmol/L to H9c2 cells	↑Trx1, Prdx2 ↑Nuclear HDAC4	[82]

Abbreviations: ADCY1: adenylate cyclase 1; ALP: Cyclophilin D; ALT: alanine transaminase; ANP, atrial natriuretic peptide; AST, aspartate aminotransferase; BNP, brain natriuretic peptide; CAT: Catalase enzyme; CK, creatine kinase; CK‐MB, creatine kinase isoenzyme; CPT1A: Carnitine palmitoyltransferase I; cTnT: Cardiac troponin; COX‐2: Cyclo‐oxygenase‐2; ERK: Extracellular signal‐regulated kinase; GSDMD: Gasdermin D; GSH, glutathione; HIF‐1; α: Hypoxia‐inducible factor 1‐alpha; HO‐1:Heme‐oxygenase 1; IFN, interferon; IL, *interleukin*; i.p., intraperitoneal; ISP, isoproterenol; IVST, interventricular septum wall thickness; JAK‐2: Janus Kinase 2; LDH, lactate dehydrogenase; LVEDP: Left ventricular end‐diastolic pressure; MDA, malondialdehyde; MMP‐9, matrix metalloproteinase‐9; MnSOD, manganese superoxide dismutase; mTOR, mammalian target of rapamycin; NF‐κB, nuclear factor kappa B; Nrf2: nuclear factor erythroid 2–related factor 2; 8‐OHdG: 8‐hydroxy‐2'‐deoxyguanosine; PKA: protein kinase A; P.o: Orally; PPAR: Peroxisome proliferator‐activated receptor; Prdx2: Peroxiredoxins; QRS: Q wave, R wave and S wave; QTc: corrected QT interval; SD, Sprague–Dawley; SIRT1, sirtuin 1; SMA, smooth muscle actin; SOD, superoxide dismutase; STAT3, signal transducer and activator of transcription 3; TNF‐α, tumor necrosis factor‐α; TRPM7, transient receptor potential melastatin 7; Trx1: Thioredoxin 1; α‐MHC, α‐myosin heavy chain.

**TABLE 4:** Plant extracts exhibited potential in ISP‐induced myocardial fibrosis.

Plant extracts and formulations	Compound dosing	ISP dosing regimen, species	Protective mechanisms	Reference
*Cymbopogon proximus* EO	800 μL/kg/day, 4 days before ISP	5 mg/kg/day for 3 days, s.c. to male albino rats	↓HW/BW, ↓ANP, BNP, and β‐MHC, collagen volume, Pro I and Pro III, cardiomyocyte degeneration, necrosis, pyknosis fraction (CVF)	[83]
*Lonicera japonica* Thunb., *Jinyinhua* *Flos Lonicerae* (Si‐Miao‐Yong, a decoction [SMYAD])	10, 20, and 40 g/kg/day p.o. for 4 weeks	5 mg/kg/day s.c. for 7 days to male SD rats	↑EF%, FS%, ↓LVESd, LVEDd, ↓HW, ↓LDH, AST, CK, CK‐MB, α‐HBDH, BNP, p‐Akt, p‐p38	[84]
*Terminalia arjuna* (Roxb.)	63, 125, and 250 mg/kg p.o.	5 mg/kg s.c. for 28 days to rats	↓HW/BW ↑Antioxidants ↓Cardiomyocyte diameter	[85]
*Dendrobium candidum*	0.13 and 0.78 g/kg/day p.o. for 1 month and 2 mg/mL 2 h before ISP	2 mg/kg/day s.c. for 10 days to SD rats and 10 μM to H9c2 cells	↓LVSP, HW/BW ↓LV/TL ↓ANP, BNP	[86]
*Panax notoginseng*	50 and 150 mg/kg, i.p. 30 min prior to ISP for 5 days	10 mg/kg, i.p. for 5 days to C57BL/6J mice	↑miR‐29c ↓*Col1a1*, *Col1a2*, *Col3a1*, *Col5a1*, *Fbn1* genes	[87]
Danshen formulae	50 mg/kg, s.c. for 7 days 1–100 μM for 30 min to NRCFs	0.25 mg/kg, s.c. for 7 days to SD rats 10 μM for 24 h to NRCFs	↓p38 MAPK ↓ROS ↑NOX2	[88]
*Gentianella acuta*	0.3, 0.6, and 1.2 g/kg, p.o. for 21 days	5 mg/kg, s.c. for 7 days to SD rats	↓TGF‐β1 expression and phosphorylation of TβRI and II	[89]
*Gentianella acuta*	0.3, 0.6, and 1.2 g/kg, p.o. on day 2 and for 21 days	5 mg/kg, s.c. for 7 days to SD rats	↓TGF‐β1, CTGF ↓NF‐κB‐P65	[90]
Blueberry extract	25, 50, and 100 mg/kg, p.o. for 28 days	5 mg/kg, s.c. for 14 days to albino rats	↓NF‐κB, COX‐2, TNF‐α, IL‐6	[91]
Citrus pectin	100 mg/kg, p.o. for 14 and 21 days	5 mg/kg, s.c. for 7 days to Wistar rats	↓Gal‐3 ↓TLR4/MyD88/NF‐κB	[92]
Chikusetsu saponin IVa (*Rhizoma Panacis japonica*)	5 mg/kg, p.o. from days 2 to 21	5 mg/kg, s.c. on day 1, followed by 2.5 mg/kg for 20 days to BALB/C mice	↓HW/BW, myocardial fibers disorderliness and atrophy, ↓inflammatory cells, ↓collagen, ↓p‐mTOR, ↓cardiomyocyte size ↓LC3β beclin‐1, ↓p62, p‐AMPK	[93]
Dangshen Erling decoction	1.28 g/kg, p.o. for 4 weeks	5 mg/kg, s.c. for 4 weeks to C57/BL6 mice	↓GM‐CSF, G‐CSF, IL‐1α, IL‐1β, IL‐3, IL‐5, IL‐7, IL‐12, IL‐13, TNF‐α, TLR4, MMP‐9, MyD88, and NF‐κB	[94]
Huoxin pill	10 and 30 mg/kg, i.p. for 7 weeks 80, 160, and 320 μg/mL	10 mg/kg, i.p. for 14 days to Wistar rats 10 μM for 24 h to neonatal rat CFs	↓TGF‐β/Smad ↓Myofibroblasts	[95]

Abbreviations: AMPK, adenosine 5′‐monophosphate (AMP)‐activated protein kinase; ANP, atrial natriuretic peptide; AST, aspartate aminotransferase; BNP, brain natriuretic peptide; CF: Cardiac fibroblasts; CK, creatine kinase; CK‐MB, creatine kinase isoenzyme; CVF: Cardiovascular failure; EF, ejection fraction; FS, fractional shortening; Gal‐3, galectin‐3; G‐CSF: Granulocyte colony‐stimulating factor; GM‐CSF: Granulocyte‐macrophage colony‐stimulating factor; IL, *interleukin*; i.p., intraperitoneal; ISP, isoproterenol; LDH, lactate dehydrogenase; LVEDd: Left ventricle enddiastolic diameter; LVESd: Left ventricle endsystolic diameter; MAPK, mitogen‐activated protein kinase; MMP‐9, matrix metalloproteinase‐9; mTOR, mammalian target of rapamycin; NF‐κB, nuclear factor kappa B; NOX2: NADPH oxidase; NRCF: neonatal rat cardiac fibroblasts; SD, Sprague–Dawley; TLR4, toll‐like receptor 4; TNF‐α, tumor necrosis factor‐α; α‐HBDH, α‐hydroxybutyrate dehydrogenase; β‐MHC, β‐myosin heavy chain.

**TABLE 5:** Pharmaceutical agents exhibited potential in ISP‐induced myocardial fibrosis.

Compound	Compound dosing	ISP dosing regimen, species	Protective mechanisms	Reference
Melatonin and omega‐3 fatty acid	Melatonin: 40 μg/mL/day Omega‐3 fatty acid: 1.68 g/kg/day in drinking water for 67 days	7–30 mg/kg s.c. for 7 days to male Wistar rats	↓Coronary flow ↓Neutrophil and macrophage infiltration ↓Collagen deposition ↓Hypertrophied tissue, ↓ activity of β‐hydroxybutyrate dehydrogenase, succinate dehydrogenase (SDH), and alkaline phosphatase, ↓Cx43 mislocalization	[96]
LCZ696 and valsartan	LCZ696 60 mg/kg/day and valsartan 30 mg/kg/day	2.4 mg/kg/day by osmotic minipump to male Wistar rats	↓Heart rate, LVSP, LVEDP, ↓Tgfb1, Clo1a1, Ccl2, Anp, NT‐proBNP	[97]
U50,488H and nor‐binaltorphimine	U50: 0.4 and 0.6 mg/kg i.p. nor‐BNI: 0.5 mg/kg for 14 days	5 mg/kg s.c. for 14 days to Wistar rats	↓HW:BW, GSH, SOD, catalase ↓Collagen ↓Necrosis ↓β‐MHC	[98]
U50,488H	1.25 mg/kg i.p. for 14 days, 10^−7^ mol/L–10^−5^ mol/L	3 mg/kg i.p. for 14 days to SD rats, 10^−6^ mol/L to neonatal rat cardiomyocytes	↓ Fibroblast proliferation ↓Collagen I, III, fibronectin, l‐type Ca^2+^ current, Ca^2+^ transient	[99]
	25 mg/kg, i.p. for 14 days	10 mg/kg, s.c. for 14 days to mice	↓Myocardial fibers, Bax, bFGF, ANP, BNP, c‐fos, c‐jun, and α‐MHC, NF‐κB (p65) and TLR4	[42]
CGEN‐856S	90 mg/kg by osmotic pump for 7 days	2 mg/kg/day, s.c. for 7 days to male Wistar rats	↓Fibronectin, collagen I and III, ↓Cardiomyocyte diameter	[100]
AS605240, a PI3Kγ inhibitor	50 mg/kg/day i.p. for 14 days	10 mg/kg for 3 days and 5 mg/kg for another 11 days s.c. to Wistar rats	↓HW/BW ↓Collagen	[101]
Ghrelin	100 μg/kg, twice daily	20, 10, and 5 mg/kg on days 1, 2, and 3, and 3 mg/kg s.c. for the next 7 days to male SD rats	↓HW, ↓irradiated ghrelin, ↓growth hormone secretagogue receptor	[102]
Phosphocreatine	200 mg/kg/day, i.p. for 45 days	50 mg/kg, twice a day, s.c. for 14 days to male SD rats	↓PWT, IVST, EF, LVDd, LVDs, and FS ↓Mass of the heart and left ventricle ↓Collagen I and III, laminin, TGF‐β1, α‐SMA, p‐p38, p‐ERK, and p‐JNK, NF‐κB, MMP‐9, TIMP‐1	[103]
Relaxin‐2	30 mg/kg, s.c. for 14 days	15 mg/kg/day by minipump for 14 days to C57BL6J mice	↓TGF‐β1/Smad, ↓protein kinase B/endothelial NO synthase, ↓protein *S*‐nitrosylation, ↑collagen	[104]
C188‐9	50 mg/kg, i.p. for 21 days	20 mg/kg by osmotic minipump for 21 days to C57BL/6 mice	↓TGF‐β1, Col1a1, Col1a2, Col3a13, α‐SMA ↓HW/BW, HW/TL ↓p‐STAT3	[105]
CTRP12	0.2 μg/g by osmotic minipump for 2 weeks	10 mg/kg for 3 days, then 5 mg/kg/day for 11 days, s.c. for 2 weeks to C57/B6 mice	↓p‐P38, p‐ERK, p‐JNK ↓CTGF ↓Fibroblast activation	[106]
KDM5B inhibitor TK‐129	20 and 50 mg/kg, p.o. twice for 24 days	5 mg/kg, i.p. for 24 days to C57BL/6 mice	↓KDM5B ↓Wnt3a, β‐catenin, Wnt5a, p‐ERK1/2, p‐JNKs	[107]
Trimetazidine	15 mg/kg, p.o. for 21 days	5 mg/kg, s.c. for 7 days to SD rats	↓EndMT ↓NOX	[108]
Pirfenidone	200 mg, p.o. for 14 days	5 mg/kg, s.c. for 7 days to C57BL/6 mice	↓JAK‐2/STAT3	[109]
Atglistatin	2 mmol/kg diet for 3 weeks	30 μg/g by osmotic minipump for 2 weeks to C57Bl/6N mice	↓Galectin‐3 positive macrophages ↓Galectin‐3	[110]
Nicorandil	5 mg/kg, p.o. for 4 weeks	5 mg/kg, s.c. for 7 days to SD rats	↑Cx43	[24]

Abbreviations: ANP, atrial natriuretic peptide; BNP, brain natriuretic peptide; CTGF: Connective Tissue Growth Factor; Cx43, connexins 43; EF, ejection fraction; EndMT, endothelial‐to‐mesenchymal transition; FS, fractional shortening; GSH, glutathione; i.p., intraperitoneal; ISP, isoproterenol; IVST, interventricular septum wall thickness; JNK: Jun N‐terminal kinase; LVDd, left ventricular end‐diastolic dimension; LVDs, left ventricular end‐systolic dimension; LVEDP: Left ventricular end‐diastolic pressure; LVSP: Left Ventricle Systolic Pressure; MHC, myosin heavy chain; MMP‐9, matrix metalloproteinase‐9; NF‐κB, nuclear factor kappa B; PWT, posterior wall thickness; SD, Sprague–Dawley; SDH: Succinate dehydrogenase; SMA, smooth muscle actin; SOD, superoxide dismutase; STAT3, signal transducer and activator of transcription 3; TLR4, toll‐like receptor 4.

Despite the enormous advantages of easy accessibility and safety profile and naturality, the challenges involved in obtaining those natural products require specific consideration before their application in clinical trials. The extraction and purification of a pure bioactive compound from herbal plants is considered a lengthy and convoluted process, and usually it is difficult to extract a 100% pure component at the end of the process, resulting in undesired imprecision. This is in addition to the need to determine the extraction methodology before extracting it as different methods have varied impact on the production purity and composition proportion. . An additional concern is that the application of the herbal plant extract to cardiac fibrosis is far from enough as its bioavailability, bioactivity, target specificity, side effects, and metabolism are still obscure and require further investigation for ensuring accurate and safe drug administration.^178^

## 5 PHARMACEUTICALS EXHIBITED POTENTIAL IN ISP‐INDUCED CARDIAC FIBROSIS

The identification of the optimal therapeutic window for targeting cardiac fibrosis will be of paramount benefit in treating it. A key therapeutic concern for antifibrosis remedies is the maturity and stiffeness of the extracellular matrix proteins  reaching to a no‐return point where ECM is densely cross‐linked and hardly degradable. In this regard, developed therapeutic agents should have the potential in targeting fibroblast activation and limiting the deposition of ECM components on cardiomyocytes and enhancing the turnover of the deposited matrix. There is still a need to pursue novel therapeutic approaches targeting fibroblasts and cardiac remodeling.

Much research effort is being devoted to exploring promising therapeutic targets as well as pharmaceutical compounds with expanding existing classical regulators of cardiac fibrosis or repurposing other agents that work at various cellular systems involved in fibrosis mediation. Table 5 presents pharmaceutical compounds that are approved for clinical use or others that are still in the preclinical stage that have been experimentally proven to target myofibroblasts and pathological cardiac remodeling cascades promoting and advancement in the treatment of cardiac fibrosis. The protective mechanisms of most of the investigated compounds include fibroblast homeostasis, TGF‐β/SMAD signaling dephosphorylation, and reversal of other signaling pathways that are implicated in collagen dysregulation. Pirfenidone is a medicine approved by the U.S. Food and Drug Administration for the treatment of pulmonary fibrosis. Current research is seeking to possibly repurpose pirfenidone to target the treatment of cardiac fibrosis based on the idea that the induction of fibrosis in different organs is fundamentally the same. In animal models of cardiac fibrosis, pirfenidone was shown to reduce atrial fibrosis and limit the expansion of induced fibrosis.^179^ In a model of ISP‐induced cardiac fibrosis, pirfenidone inhibited the activation of transcription factors that ultimately eliminate the production of fibrotic modulators. Further research should be directed to detect beneficial therapeutic effects that can protect against and mitigate cardiac fibrosis.

## 6 GENETIC, SURGICAL, AND ISP‐INDUCED MODELS OF CARDIAC FIBROSIS

There are various models that rely on genetic modification of animals to induce fibrosis. Genetic models are obtained using gene deletion, overexpression, or mutation. Given the clinical significance of hypertension, spontaneously hypertensive rats (SHR) are used as preclinical models of cardiac fibrosis. Hypertension is known to exacerbate the risk of heart diseases and the development of fibrosis in the long term. It is also shown to be associated with the accumulation of ECM protein on the myocardium.^180^ Hypertension starts to develop at ~6–8 weeks of age and generally plateaus to a stable value in adulthood between 3 and 5 months. It is documented that 16–20 week adult SHRs experience maximal hypertension, but cardiac fibrosis is not pathological, which limits the translational relevance of drug testing. This is, in particular, because most patients with vascular diseases are middle aged to elderly and are likely to have well‐established fibrosis prior to treatment. However, some studies have used SHRs at older ages (10–20 months), in which fibrosis has been established before drug administration, which is more clinically relevant. Another model is the transgenic rat model overexpressing mouse renin (mRen‐2) gene. Renin is known for its role in increasing blood pressure by converting to angiotensin I, which in turn plays a role in constricting blood vessels. In mRen‐2 overexpressed rats, the pathology is similar to SHRs, where it depends on the chronicity of hypertension; however, it is less used because of its decreased availability. Rats start to develop hypertension early at 4 weeks, and at week 10 they start to manifest cardiac hypertrophy and perivascular fibrosis (mainly collagen type I), which are associated with increased profibrotic cytokine (TGF‐β1) expression.^181^

In surgical models, the transverse aortic constriction (TAC) is mostly used for the induction of fibrosis. It depends on the elevation of the afterload by the ligation of the aortic arch resulting in left ventricular hypertrophy and tissue remodeling, associated with an increased diameter of myocardial myocytes, accumulation of intercellular collagen, and left ventricular function impairment. Studies have shown that hypertrophy and fibrosis are observed 4 weeks after the rat aortic arch is ligated.^181^ The ligation is performed by suturing against the inner aorta to create the aortic narrowing of about 60%–70%.^182^ This model is advantageous for its outcome of quantified pressure gradient across the aortic stenosis and the stratified left ventricular hypertrophy; however, the main drawback is the high early mortality rate of rats (30%) related to acute cardiac insufficiency.^183^ Constricting the ascending aorta is another model of creating pressure and increasing cardiac afterload. The mechanism in inducing cardiac fibrosis and myocardial hypertrophy is similar to TAC surgery where molecular and neurohumoral stimulatory pathways are activated to induce cardiac remodeling, with a high success rate of 80%.^184^ These types of induction are associated with a low mortality rate after operation and a highly reproducible fibrotic phenotype. However, the disadvantages include the intensive training required in addition to surgery specifications such as open chest ventilation. Other surgical models include permanent ligation of the coronary artery. The surgical models generally cause acute injury that leads to ischemia‐induced cardiomyocyte death. The coronary blockade triggers tissue necrosis that ultimately initiates the inflammatory, reparative, and maturation phases in which cardiac myofibroblasts secrete extracellular proteins to make up for the lost tissue. The scar takes ~28 days to be fully formed.^185^

On the contrary, fibrosis can be induced noninvasively chemically by the administration of ISP. Being a catecholamine analog, ISP activates β2 receptors and causes pacing‐induced heart injury. It is considered a chronotropic and inotropic agent that increases contractility force and heart rate causing heart damage. Studies have shown the variation by which ISP can be used to induce fibrosis. It can be used to induce either acute or chronic type of injury. Acute models use a high dose of ISP (85–100 mg/kg for 1–2 days) for a short duration,^186^ and chronic models follow a long‐term treatment with low doses (5–50 mg/kg daily for 1–3 weeks).^187^ The degree of the developed fibrosis varies according to the followed protocol; however, it is reported to be 4‐ to >20‐fold compared to uninjected animal necrosis peaking after 48 h of ISP injection.^188^ ISP is preferred for its ability to induce reparative fibrosis after acute induction and reactive fibrosis after chronic induction of preclinical rodent models of cardiac fibrosis.^181^

## 7 CONCLUSION

The mechanisms highlight the potential of ISP to be used as a valuable tool for both manifesting the pathogenesis associated with cardiac fibrosis and subsequently evaluating the antifibrotic effects of various pharmacological compounds. It provides a step toward an experimental model system to study the pathological changes in fibrotic hearts. ISP‐induced fibrosis in experimental models can be adapted to mimic the various aspects of cardiac fibrosis, including histological changes, biochemical alteration, and functional impairment. Therefore, it provides further insight into mechanisms underlying cardiac remodeling and contributes to the development of antifibrotic agents.

A perfect animal model system is necessary to identify the key pathologic features, pathophysiological correlates, pharmacological targets, and molecular mechanisms. A reproducible model could be the most important approach to understand pathophysiological events and offer testing agents for the prevention and treatment as well as aid diagnostic and prognostic markers. Until benchmark animal models become available, ISP‐induced myocardial fibrosis and cardiac hypertrophy could be well employed for pathological, pharmacological, and physiological studies. Although the ISP‐based model is advantageous for its wide use, reproducibility, noninvasive nature, and the ability to mimic both reactive and reparative cardiac fibrosis, where it was suggested that an acute high dose of ISP induced myocardial necrosis simulating the reparative type of fibrosis, it has some drawbacks and limitations that require consideration. ISP is used in many protocols in studies. Some used acute high doses for a couple of days, whereas others opted the chronic induction with low doses. In addition, in some cases, the effect of ISP in inducing fibrosis was not observed immediately but a few days after administration. This in turn will reflect the high variability in the extent of the developed fibrosis and the accompanied mortality rate. However, ISP‐induced cardiac injury is considered a typical preclinical model that is used for relevant therapeutic target search in the field of cardio‐pharmacology.

## AUTHOR CONTRIBUTIONS

**Lujain Bader Eddin:** Data curation; formal analysis; methodology; validation; writing – original draft; writing – review and editing. **M. F. Nagoor Meeran:** Data curation. **Niraj Kumar Jha:** Visualization. **Samer N. Goyal:** Data curation. **Shreesh Ojha:** Conceptualization; funding acquisition; project administration; supervision.

## FUNDING INFORMATION

This research received no external funding.

## CONFLICT OF INTEREST STATEMENT

The authors thank the United Arab Emirates University for theaward of the research grants (12R121 and 12R104 to Shreesh Ojha).

## Ethical Statement

None.
